# Results of a Geant4 benchmarking study for bio‐medical applications, performed with the G4‐Med system

**DOI:** 10.1002/mp.17678

**Published:** 2025-02-21

**Authors:** Pedro Arce, Jay W. Archer, Lorenzo Arsini, Alexander Bagulya, David Bolst, Jeremy M. C. Brown, Barbara Caccia, Andrew Chacon, Giuseppe Antonio Pablo Cirrone, Miguel Antonio Cortés‐Giraldo, Dean Cutajar, Giacomo Cuttone, Paolo Dondero, Andrea Dotti, Bruce Faddegon, Serena Fattori, Christian Fedon, Susanna Guatelli, Akihiro Haga, Sebastien Incerti, Vladimir Ivanchenko, Dmitri Konstantinov, Ioanna Kyriakou, Albert Le, Zhuxin Li, Michel Maire, Alessandra Malaroda, Carlo Mancini‐Terracciano, Alfonso Mantero, Claire Michelet, Giuliana Milluzzo, Francesca Nicolanti, Mihaly Novak, Chihiro Omachi, Luciano Pandola, Jake Harold Pensavalle, Álvaro Perales, Yann Perrot, Giada Petringa, Silvia Pozzi, José Manuel Quesada, José Ramos‐Méndez, Francesco Romano, Anatoly B. Rosenfeld, Mitra Safavi‐Naeini, Dousatsu Sakata, Luis G. Sarmiento, Takashi Sasaki, Yoshihide Sato, Alberto Sciuto, Ioannis Sechopoulos, Edward C. Simpson, Ronny Stanzani, Alessandra Tomal, Toshiyuki Toshito, Hoang Ngoc Tran, Christopher White, Dennis H. Wright

**Affiliations:** ^1^ CIEMAT Madrid Spain; ^2^ Centre for Medical Radiation Physics University of Wollongong Wollongong New South Wales Australia; ^3^ Sapienza, University of Rome Rome Italy; ^4^ INFN, Roma1 Section Rome Italy; ^5^ Lebedev Physical Institute Moscow Russia; ^6^ Swinburne University of Technology Melbourne Victoria Australia; ^7^ Istituto Superiore di Sanità Rome Italy; ^8^ Australian Nuclear Science and Technology Organisation Lucas Heights New South Wales Australia; ^9^ INFN LNS Catania Italy; ^10^ Centro Siciliano di Fisica Nucleare e Struttura della Materia Catania Italy; ^11^ Universidad de Sevilla Sevilla Spain; ^12^ SWHARD srl Genova Italy; ^13^ SLAC National Accelerator Laboratory Stanford California USA; ^14^ University of California San Francisco California USA; ^15^ Nuclear Research and Consultancy Group (NRG) LE Petten The Netherlands; ^16^ Tokushima University Tokushima Japan; ^17^ CNRS, Univ. Bordeaux, LP2I Bordeaux, UMR5797 Gradignan France; ^18^ Tomsk State Research University Tomsk Russia; ^19^ University of Virginia Charlottesville Virginia USA; ^20^ Ioannina University Ioannina Greece; ^21^ LAPP, IN2P3 Annecy France; ^22^ Medical Imaging Department Nepean Blue Mountains LHD Sydney New South Wales Australia; ^23^ INFN Catania Division Catania Italy; ^24^ CERN Geneva Switzerland; ^25^ Nagoya Proton Therapy Center Nagoya Japan; ^26^ Azienda Ospedaliera Universitaria Pisa Pisa Italy; ^27^ Hospital Universitario Puerta de Hierro Majadahonda Spain; ^28^ IRSN Fontenay‐aux‐Roses France; ^29^ Osaka University Osaka Japan; ^30^ Department of Physics Lund University Lund Sweden; ^31^ KEK Tsukuba Japan; ^32^ Radboud University Medical Center Nijmegen The Netherlands; ^33^ Dutch Expert Center for Screening (LRCB) Nijmegen The Netherlands; ^34^ Department of Nuclear Physics and Accelerator Applications Research School of Physics Australian National University Canberra Australia; ^35^ Universdade Estadual de Campinas (UNICAMP) Campinas Brazil

**Keywords:** benchmarking, bio‐medical physics, Geant4

## Abstract

**Background:**

Geant4, a Monte Carlo Simulation Toolkit extensively used in bio‐medical physics, is in continuous evolution to include newest research findings to improve its accuracy and to respond to the evolving needs of a very diverse user community. In 2014, the G4‐Med benchmarking system was born from the effort of the Geant4 Medical Simulation Benchmarking Group, to benchmark and monitor the evolution of Geant4 for medical physics applications. The G4‐Med system was first described in our Medical Physics Special Report published in 2021. Results of the tests were reported for Geant4 10.5.

**Purpose:**

In this work, we describe the evolution of the G4‐Med benchmarking system.

**Methods:**

The G4‐Med benchmarking suite currently includes 23 tests, which benchmark Geant4 from the calculation of basic physical quantities to the simulation of more clinically relevant set‐ups. New tests concern the benchmarking of Geant4‐DNA physics and chemistry components for regression testing purposes, dosimetry for brachytherapy with a 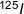 source, dosimetry for external x‐ray and electron FLASH radiotherapy, experimental microdosimetry for proton therapy, and in vivo PET for carbon and oxygen beams. Regression testing has been performed between Geant4 10.5 and 11.1. Finally, a simple Geant4 simulation has been developed and used to compare Geant4 EM physics constructors and physics lists in terms of execution times.

**Results:**

In summary, our EM tests show that the parameters of the multiple scattering in the Geant4 EM constructor *G4EmStandardPhysics_option3* in Geant4 11.1, while improving the modeling of the electron backscattering in high atomic number targets, are not adequate for dosimetry for clinical x‐ray and electron beams. Therefore, these parameters have been reverted back to those of Geant4 10.5 in Geant4 11.2.1. The x‐ray radiotherapy test shows significant differences in the modeling of the bremsstrahlung process, especially between *G4EmPenelopePhysics* and the other constructors under study (*G4EmLivermorePhysics*, *G4EmStandardPhysics_option3*, and *G4EmStandardPhysics_option4*). These differences will be studied in an in‐depth investigation within our Group. Improvement in Geant4 11.1 has been observed for the modeling of the proton and carbon ion Bragg peak with energies of clinical interest, thanks to the adoption of ICRU90 to calculate the low energy proton stopping powers in water and of the Linhard–Sorensen ion model, available in Geant4 since version 11.0. Nuclear fragmentation tests of interest for carbon ion therapy show differences between Geant4 10.5 and 11.1 in terms of fragment yields. In particular, a higher production of boron fragments is observed with Geant4 11.1, leading to a better agreement with reference data for this fragment.

**Conclusions:**

Based on the overall results of our tests, we recommend to use *G4EmStandardPhysics_option4* as EM constructor and *QGSP_BIC_HP* with *G4EmStandardPhysics_option4*, for hadrontherapy applications. The Geant4‐DNA physics lists report differences in modeling electron interactions in water, however, the tests have a pure regression testing purpose so no recommendation can be formulated.

## 1 INTRODUCTION

Geant4^1^, ^2^, ^3^ is an open source Monte Carlo Simulation Toolkit modeling particle interactions and transport in matter. Geant4 has undergone continuous development and enhancement since it was initially made available to the public in 1998. These improvements have expanded the software's functionality to include newest research findings to improve its accuracy and allowed it to respond to the evolving needs of a very diverse user community, which includes researchers in fields such as high‐energy physics, space, nuclear, and bio‐medical sciences. The software is developed by an international scientific Collaboration, based at CERN, Geneva, Switzerland, counting more than 100 contributors worldwide. The Monte Carlo code is released with a new public version every year in December. Prior to this, a beta version is usually released in June, and subsequent patches may be issued to fix errors.

**FIGURE 1:**
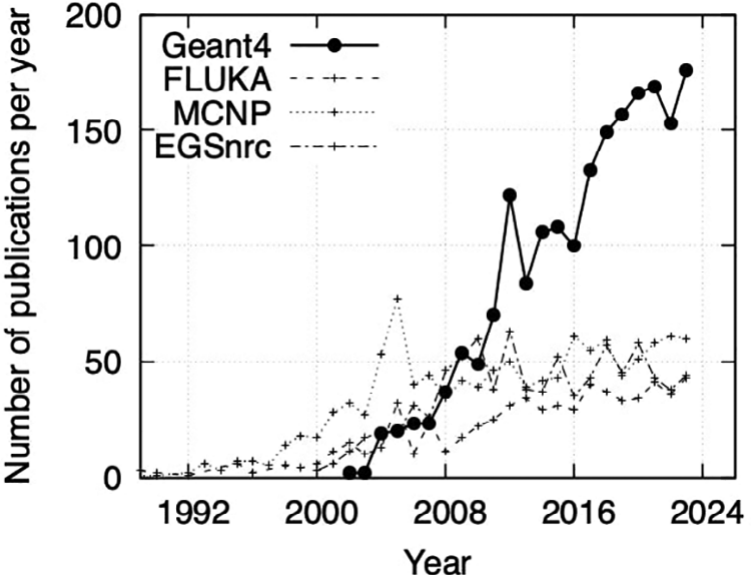
Number of scientific publications in bio‐medical physics found in the PubMed Central®^5^ archive, searching the general purpose Monte Carlo codes Geant4,^1^, ^2^, ^3^ and FLUKA,^6^, ^7^, ^8^ and MCNP^9^ and EGSnrc^10^, ^11^ in keywords, titles and citations of papers recorded in the PubMed archive. The figure contains data (up to 2018) published in Mancini‐Terracciano et al.,^12^ Copyright Elsevier (2019).

As shown in Figure 1, since its first release, Geant4 has become increasingly popular in the bio‐medical physics community. Given its extensive use in the scientific community and its wide set of physics models offered to describe both electromagnetic and hadronic physics interactions, in 2014 G4‐Med, the first Geant4 benchmarking system for bio‐medical physics applications was established. It was born as an effort of the Geant4 Medical Simulation Group^4^ to benchmark different physics models of Geant4 in a set of exemplary bio‐medical application scenarios of interest.

This international effort was documented for the first time in Arce et al.^13^ The tests, ranging from fundamental physical quantities to more realistic applications, were developed and executed with Geant4 10.5 on the CERN computing infrastructure, via the *geant‐val* system.^14^, ^15^

In this special report, we document the evolution of the G4‐Med benchmarking system in terms of new tests that have been included and tests that have been revised. We then report the results of the regression tests performed with Geant4 versions 10.5 and 11.1 (released in December 2022), which was the most recent release of Geant4 when the study described in this special report was started. Finally, we document for the first time the benchmarking of the execution times of the G4‐Med tests when using different Geant4 physics lists and constructors. The latter is a critical information in those Geant4‐based studies where the computing performance is an important factor.

## 2 METHODOLOGY

The G4‐Med benchmarking system, documented for the first time in Arce et al.,^13^ currently includes 23 tests, listed in Table 1 and covers different bio‐medical application scenarios of interest. The tests are designed to benchmark Geant4 from the calculation of fundamental physical quantities to the simulation of more clinically relevant set‐ups modeling a medical use of ionizing radiation. The tests in Table 1 are categorized according to the specific physics models and interactions being tested. These categories include electromagnetic physics tests (Section 3), tests for hadronic nuclear cross sections (Section 4), tests for both electromagnetic and hadronic physics (Section 5) and Geant4‐DNA tests (Section 6).

**TABLE 1:** Tests of *G4‐Med* with their name in the *geant‐val* interface and the subsections of this report with their description and results.

Test	Name in *geant‐val* interface^15^	Source	Section
**Electromagnetic physics tests**			
**Brachytherapy** 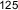 **I and** 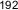 **Ir sources**	*Brachy*	^b^	3 and 3.2
**MV x‐ray radiotherapy**	*medical_linac*	^b^	3.3
**Electron FLASH radiotherapy**	*eFLASH_radiotherapy*	^b^	3.4
Electron backscattering	*ElectBackScat*	Dondero et al.^16^	3.5.1
13‐MeV electron forward scatter from foils	*ElecForwScat*	Faddegon et al.^17^	3.5.2
Bremsstrahlung from thick targets	*Bremsstrahlung*	Faddegon et al.^18^	3.5.3
Fano cavity	*FanoCavity*	^a^	3.5.4
Photon attenuation	*PhotonAttenuation*	Amako et al.^19^	3
Electron electronic stopping power	*ElectDEDX*		3
Monoenergetic x‐ray internal breast dosimetry	*Mammo*	Fedon et al.^20^, ^21^	3
**Hadronic nuclear cross section tests**			
Nucleus–Nucleus hadronic inelastic scattering cross sections	*NucNucInelXS*		4.1.1
62 MeV/u 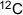 fragmentation	*LowEC12Frag*	Mancini‐Terracciano et al.^22^	4.1.2
Charge‐changing cross section for 300‐MeV/u carbon ions	*C12FragCC*	Toshito et al.^23^	4.1.3
**Electromagnetic and hadronic physics tests**			
**62‐MeV proton beam test: cell survival modeling and track‐averaged LET**	*Hadrontherapy*	^b^, Petringa et al.^24^, ^25^	5.2
**In vivo PET for carbon ion therapy**	*heavy_ion_therapy*	Chacon et al.^26^, ^27^	5.3
67.5‐MeV proton Bragg curves in water	*LowEProtonBraggBeak*	Faddegon et al.^28^	5.4.1
Light ion Bragg peak curves	*LightIonBraggPeak*		5.4.2
Neutron yield of protons with energy 113 and 256 MeV and 290‐MeV/u carbon ions	*ProtonC12NeutronYield*		5.4.3
Fragmentation of a 400‐${\mathrm{MeV}/u}^{12}C$ ion beam in water	*FragTest*	Bolst et al.^29^	5.4.4
**Geant4‐DNA tests**			
**Low energy electron**	*LowEElectDPK*	^a^	6.3
**Dose Point Kernels**			
**Microdosimetry**	*microyz*	^a^, Kyriakou et al.^30^	6.4
**Chemistry**	*chem6*	^a^	6.5

*Note*: New tests (introduced in the G4‐Med benchmarking suite since 2021) are in bold. All the other tests have been documented in detail in Arce et al.^31^ The Geant4‐DNA and Fano cavity tests have a purely regression testing purpose.

^a^ Tests released as Geant4 *extended* examples.

^b^ Tests released as Geant4 *advanced* examples.

New tests have been included to benchmark Geant4 for dosimetric calculations in water phantoms, for brachytherapy with an 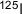 source (*Brachytherapy* test), external MV x‐ray radiotherapy (*MV x‐ray radiotherapy* test), and electron FLASH radiotherapy (*electron FLASH radiotherapy* test).

A new hadronic physics test benchmarks Geant4 for in vivo positron emission tomography (PET) for carbon ion therapy (*in vivo PET for carbon ion therapy* test), while the *62 MeV proton beam* test, originally developed for the calculation of cell survival curves irradiated with a 62 MeV spread out Bragg peak proton beam, has been extended to benchmark the track‐averaged LET against experimental measurements.^24^

The *Microdosimetry* test and the *Low energy electron Dose Point Kernels* test, described in Arce et al.,^13^ have been adapted to benchmark Geant4‐DNA physics lists, while the *Chemistry* test has been introduced to benchmark different Geant4 models to describe the chemical stage of particle interactions in water.

The G4‐Med benchmarking system runs all its tests in the *geant‐val* environment.^14^, ^15^ The *Chemistry* test is executed on the computing facility of the Laboratoire de Physique des Deux Infinis Bordeaux (LP2IB), while the other tests are executed on the CERN computing infrastructure. Tests can be executed whenever it is necessary to verify the functionality of Geant4 and support the development of Geant4 for bio‐medical physics applications.

The new tests of the G4‐Med benchmarking suite (in bold in Table 1) have been executed only with Geant4 11.1 as they were integrated in the system after the release of Geant4 11.0. Provided the differences in terms of kernel of Geant4 between version 10.5 and 11.1, it would be very impractical to maintain the tests for older versions of Geant4, while the maintenance is done systematically for newer versions of the Toolkit. Regression testing between Geant4 10.5 and 11.1 has been done for all the tests that were already integrated in the G4‐Med system when Geant4 10.5 was the most recent version of the Monte Carlo code. All comparisons against reference data are performed with a 95% confidence level.

When the tests are released as extended or advanced examples of Geant4, apart from the case of the *Electron FLASH radiotherapy* test, the user can repeat the test locally with either the same or different simulation parameters. The exception of the *Electron FLASH radiotherapy* test is due to the fact that the beamline details, used in the test, cannot be released publicly with Geant4 because of nondisclosure agreements with third parties. Therefore, the results obtained with the advanced example may be different from those documented here.

The tests are summarized in the website dedicated to the G4‐Med project,^4^ while the simulation results documented in this special report can be downloaded from the *geant‐val* web interface.^15^

### 2.1 Regression testing method

Regression testing between Geant4 versions 10.5 and 11.1 is performed to benchmark how the evolution of Geant4 and its physics capability affects the results of the G4‐Med tests. The tests are executed via *geant‐val* with the two different Geant4 versions, using the same simulation configuration and user‐defined physics parameters (for example, *cut* and *maximum step*). The parameters' space is the same when comparing Geant4 10.5 and 11.1.

The results of the Geant4 regression testing are compared to the reference data, in the tests where such data exist. If the reference data are not available (see *Low energy electron Dose Point Kernels* and *microdosimetry* tests), the Geant4 models are compared to quantify the impact of their modeling differences on physical quantities of interest.

The ratios r of the simulation results S, obtained with either Geant4 10.5 or 11.1, and the reference data R, that is, $r_{10.5}=\frac{S_{10.5}}{R}$ and $r_{11.1}=\frac{S_{11.1}}{R}$, are computed for each point of the dataset. If there is a small misalignment along the *x*‐axis in the points at which the simulated and reference data points are evaluated, the closest simulated data point is taken in the computation of the ratio.

The agreement between simulation results and reference data is calculated with a confidence level of 95%. If the calculated ratios, $r_{10.5}$ and $r_{11.1}$, agree with 1 within their 2σ value, defined as $\sigma =r_{Geant4\_version}\times \sqrt{{\left(\frac{\sigma_{i,ref}}{R_{i}}\right)}^{2}+{\left(\frac{\sigma_{i,sim}}{S_{i}}\right)}^{2}}$, where $S_{i}$, $R_{i}$, $\sigma_{i,sim}$, and $\sigma_{i,ref}$ are the individual data points of the simulation results and reference data and associated uncertainties, respectively, then it is concluded that both Geant4 10.5 and 11.1 agree with the reference data. If instead, it is found that a release of Geant4 does not agree with 1 within 2σ, the difference is deemed significant. In this case, the 10.5 and 11.1 releases of Geant4 are compared in terms of the mean relative error (MRE), the normalized mean absolute error (NMAE), and the maximum difference (MD) with the reference data to quantify which release of Geant4 produces results closer to the reference data. The uncertainties affecting these metrics correspond to 1σ as we intend to have a more restrictive comparison in terms of regression testing between the two versions of Geant4.

The MRE is calculated as follows:

(1) $${\mathrm{MRE}}_{\mathrm{Geant}4\_\mathrm{version}}=\frac{1}{n}\left[\sum_{i=1}^{n}\frac{|S_{i}-R_{i}|}{R_{i}}\right]\;$$

where $S_{i}$ and $R_{i}$ are the individual data points of the simulation results and reference data, respectively, while *n* is the number of the data points of the distribution under testing.

The NMAE as defined in Equation (2) is also considered to describe the absolute deviation of simulations to the reference data.

(2) $${\mathrm{NMAE}}_{\mathrm{Geant}4\_\mathrm{version}}\equiv \frac{\frac{1}{n}\sum_{i}\left|S_{i}-R_{i}\right|}{\frac{1}{n}\sum_{i}R_{i}}=\frac{\sum_{i}\left|S_{i}-R_{i}\right|}{\sum_{i}R_{i}}.$$

Analogously to the calculation of the MRE, $S_{i}$ and $R_{i}$ are the individual data points of the simulation results and reference data, respectively, and *n* is the number of the data points of the distribution under testing.

To complete the analysis, the MD between the simulation and the reference data, shown in Equation (3), is calculated for each Geant4 release under study to demonstrate the most extreme differences from the experimental data.

(3) $${\text{MD}}_{\mathrm{Geant}4\_\mathrm{version}}=\max\left|S_{i}-R_{i}\right|\;,$$

where $S_{i}$ and $R_{i}$ are the individual data points of the simulation results and reference data, respectively.

Regression testing results are reported and commented on in this article only when there are significant differences (more than 2σ with the reference data) for any of the two releases of Geant4. Nevertheless, the complete data analysis performed in this work can be accessed through the G4‐Med analysis website.^32^

### 2.2 Computing performance

An important consideration when choosing a Geant4 physics list or constructor for a specific simulation scenario is first the accuracy of results, and then the execution time. If a physics list is more accurate than another, compared to reference/experimental data, but significantly more computationally intensive, then the user may need to decide whether the added accuracy is required or if the added computing resources are justifiable.

The aim of this part of the project, documented in Section 7, is to benchmark the execution times of the Geant4 physics constructors and lists under testing in the G4‐Med suite using a simple Geant4 simulation application.

## 3 ELECTROMAGNETIC PHYSICS BENCHMARKING TESTS

The electromagnetic (EM) physics tests listed in Table 1 have been executed with Geant4 11.1 via the *geant‐val* system. Following the methodology described in Arce et al.,^13^ the EM physics constructors under study are *G4EmLivermorePhysics*, *G4EmPenelopePhysics*, *G4EmStandardPhysics_option3*, and *G4EmStandardPhysics_option4*, called here *Livermore*, *Penelope*, *Opt3*, and *Opt4*, respectively. The EM physics constructor *G4EmStandardPhysicsSS*, called here *SS*, is considered in the *electron backscattering* test (see Section 3.5.1) as it uses the single scattering model to describe the Coulomb scattering. The EM constructor *G4EmStandardPhysics* has been excluded from the benchmarking study as our previous work^13^ demonstrated that it is not appropriate for bio‐medical applications.

The EM physics constructors under study have been documented in Arce et al.^13^ and their evolution between Geant4 10.5 and 11.1 is reported in Section 3.1. Sections 3.2, 3.2.1, 3.2.2, 3.3, 3.3.1, 3.3.2, 3.4 describe the new tests, *brachytherapy* with an 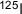 source, *MV x‐ray radiotherapy*, and *Electron FLASH radiotherapy*, respectively. Section 3.5 reports significant findings resulting from the regression testing of all the other tests, described in Arce et al.,^13^ between Geant4 10.5 and 11.1.

### 3.1 Evolution of the Geant4 electromagnetic physics constructors under study

*Livermore*, *Penelope*, *Opt3*, and *Opt4* EM constructors correspond to different combinations of models deriving from either Geant4 *Standard*, *Livermore* or *Penelope* packages (Geant4 Physics Reference Manual^13^, ^33^). For a detailed description of the EM constructors, the reader can refer to Arce et al.,^13^ while here only significant revisions between Geant4 10.5 and 11.1 are commented.

Table 2 lists the Geant4 11.1 configuration of processes and corresponding models in each EM constructor studied in this work. Since Geant4 11.1, the *Livermore* physics processes use by default the newly introduced EPICS2017 (Electron–Photon Interaction Cross Sections)^34^, ^35^, ^36^ data libraries to describe Rayleigh scattering, photoelectric effect, Compton scattering, and gamma conversion processes. The EPICS2017 models of Rayleigh scattering and photoelectric effect are also used in the *Opt3* and *Opt4* constructors. The *G4BetheHeitler5DModel* ^37^, ^38^ is used for the sampling of the final state of gamma conversion to electron–positron pair in *Opt3*, *Opt4*, and *Livermore* EM configurations. In Geant4 11.1, in *Opt4*, the Penelope model substitutes the Livermore model to describe the ionization process of electrons with energy below 100 keV.

**TABLE 2:** Geant4 physics models to describe EM physics processes up to 1 GeV, in the Geant4 EM constructors under investigation.

Geant4	*WVI*	*Opt3*	*SS*	*Opt4*	*Livermore*	*Penelope*
Rayleigh scattering and photoelectric effect	**Livermore (EPICS2017)** Li et al.^36^					PENELOPE
Compton scattering	*G4KleinNishina Model*			*G4LowEPComptonModel* for E < 20 MeV Brown et al.,^39^ *G4KleinNishina* for E >20 MeV	**Livermore (EPICS2017)** Li et al.^36^	PENELOPE
Gamma conversion	***G4BetheHeitler5DModel*** Bernard^37^, ^38^				***G4Livermore5DModel*** Li et al.^36^	PENELOPE
$e^{-}$ and $e^{+}$ ionization	Standard			**PENELOPE for E** < **100 keV**, Standard for E > 100 keV	Livermore for E < 100 keV Standard for E > 100 keV	PENELOPE
$e^{-}$ and $e^{+}$ bremsstrahlung	Geant4 *Standard* Model	*G4SeltzerBergerModel* for E < 1 GeV, *G4eBremsstrahlungRelModel* for E > 1 GeV				PENELOPE
$e^{+}$ annihilation	*G4eplusTo2GammaOKVIModel* ^33^	Standard				PENELOPE
$e^{-}$ and $e^{+}$ multiple scattering	Urban model for E < 1 MeV, Wentzel model for E > 1 MeV	Urban model	Not available	Goudsmit‐Saunderson model^40^, ^41^ for E < 100 MeV Wentzel model for E > 100 MeV		
Coulomb scattering	on	off	on			
Bremsstrahlung angular distribution	*Modified Tsai*	*2BS*	*Modified Tsai*	*2BS*		PENELOPE

*Note*: Geant4 11.1 is considered. For details on the models, the reader should refer to the Geant4 Physics Reference Manual.^33^ Changes with respect to Geant4 10.5 are shown in bold.

All the EM constructors use the same EM physics models for protons. The only change between Geant4 10.5 and 11.1 is the adoption of ICRU90 stopping power data for water, air, and graphite. For the other materials, NIST PSTAR data are used if available. If not, stopping powers are computed using ICRU49 evaluated data. A novel feature in Geant4 11.1 is that all the EM constructors, tested in this report, include a new ionization model for ions heavier than Helium, the *G4LindhardSorensenIonModel*.^33^ In this model, the ICRU90 and ICRU73 data for stopping power are used below 2 MeV/u. The mean excitation energy of water *I* is equal to 78 eV, as recommended by the ICRU Report 90^42^ in both Geant4 10.5 and 11.1.

Table 3 reports multiple scattering parameters and other options. Differences between Geant4 10.5 and 11.1 are highlighted in bold. A significant change between Geant4 10.5 and 11.1 is that in *Opt3*, the *RangeFactor* has been changed from 0.04 to 0.03 and that the use of step limitation algorithm for multiple scattering^43^ *UseDistanceToBoundary* has been substituted with *UseSafetyPlus*, with the intent of providing faster simulation, more robust tracking in magnetic field and more accurate simulation results for electron and positron backscattering. In the *SS* EM constructors, the use of the step limitation algorithm for multiple scattering *UseDistanceToBoundary* has been substituted with *UseSafetyPlus*.

**TABLE 3:** Geant4 EM parameters (described in Arce et al.^13^ and in the Geant4 Physics Reference Manual^33^) of the EM constructors under investigation.

Geant4 EM parameter	*Opt3*	*Opt4 Livermore Penelope*	*SS*
*Minimum energy* (eV)	10	**100**.	100
*Lowest electron energy* (keV)	0.1	0.1	0.01
*Number of bins per decade*	20	20	7
*Mott corrections*	on	on	on
*dRoverRange* for $e^{-}$ and $e^{+}$	0.2	0.2	0.2
*finalRange* for $e^{-}$ and $e^{+}$ (mm)	0.1	**0.01**	1
*dRoverRange* for muons and hadrons	**0.2**	**0.1**	**0.2**
*finalRange* (mm) for muons and hadrons	0.05	0.05	0.1
*Skin for e* 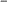 and $e^{+}$	1	3	1
*Range factor* for $e^{-}$ and $e^{+}$	**0.03**	0.08	0.04
*Range factor* for muons and hadrons	0.2	0.2	0.2
Msc StepLimitType	***fUseSafetyPlus*** ^a^		
Fluorescence and Auger $e^{-}$	On		
PIXE modeling	Off	Off	On

*Note*: Geant4 11.1 is considered. Changes with respect to Geant4 10.5 are shown in bold.

^a^ The use of *fUseSafetyPlus* is a new feature for the EM constructor *Opt3* and *SS*.

### 3.2 Brachytherapy test— source

Since the publication of Arce et al.,^13^ the *Brachytherapy* test, which is released with Geant4 as an advanced example, has been extended to calculate the radial dose rate distribution of an ${\text{Oncura}}^{TM}$ brachytherapy 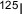 Model 6711 source in water. The test has been included in the *geant‐val* interface as an option of the *Brachytherapy* test. The results of the simulation are compared with reference data published in Dolan et al.,^44^ which are experimental measurements performed with TLD detectors.

#### 3.2.1 Simulation set‐up

The Oncura^TM^ Model 6711, described in Dolan et al.,^44^ has been implemented in the Geant4 simulation. The 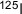 source emission is obtained by modeling the radioactive decay of iodine with the Geant4 *General Particle Source*. The iodine radioactive core is a cylinder with 0.247 mm radius and the length of the cylinder is 2.794 mm. These lengths take into account that the radioactive coating is implanted 3 μm deep onto the surface of the silver core. The 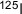 source is modeled in the center of a water box (modeled as G4_WATER) with 30 cm size. The energy deposition is scored with a Geant4 scoring mesh with voxels 0.25 mm wide and the threshold of production of secondary particles is set equal to 0.05 mm. The radial dose rate distribution is calculated as the energy deposition per unit of mass, along the transverse axis of the source, 90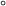 from the source axis.

10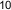 histories are executed to obtain a statistical uncertainty lower than 1% for distances up to 10 cm from the brachytherapy source. The statistical uncertainty was determined by executing 10 simulations with different random seeds and consisting each of 10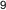 histories and evaluating the standard error affecting the average dose values.

The reference data are experimental measurements performed with thermoluminescent dosimeters, which are documented in Dolan et al.^44^

#### 3.2.2 Results and discussion

**FIGURE 2:**
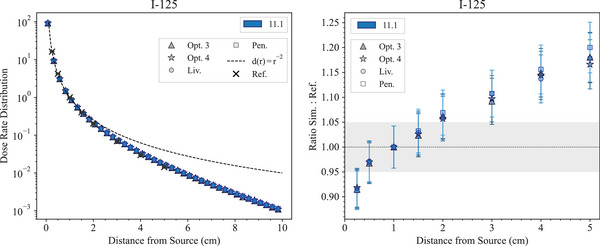
Left: Radial dose rate distribution with respect to the distance from the source, calculated with the *Brachytherapy* test in the case of an 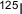 Oncura^TM^ 6711 source. When not visible, the error bars are within the symbols. The dashed line represents the inverse square law distribution as is the case for a non interacting isotropic point source. Right: Ratio of the simulation and experimental data (Dolan et al.^44^). The shadowed area represents an agreement within 5% with the reference data. Geant4 11.1 is used. To note, the two plots have different scales on the *x*‐axis.

Figure 2 shows the radial dose rate distribution in water produced by the 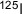 brachytherapy source, calculated with Geant4 11.1 and compared to the reference data. The agreement between the Geant4 simulation results and the reference data is within 2σ for distances between 0.5 cm and approximately 2 cm. The agreement is within 10% up to 3 cm and within 20% up to 5 cm. All the Geant4 EM constructors under study provide the same level of agreement with the reference data.

### 3.3 MV x‐ray radiotherapy test

The widespread use of Monte Carlo simulations in radiotherapy has led us to evaluate the impact of the Geant4 EM physics constructors under study when simulating a linear accelerator model for external x‐ray radiotherapy. The *MV x‐ray radiotherapy* test is based on the Geant4 advanced example *medical_linac* and is validated with experimental data. The reference data were derived from the intercomparison of different Monte Carlo simulation codes for the modeling of a medical linear accelerator, conducted by the Computational Dosimetry Working Group of EURADOS.^45^, ^46^

The linear accelerator implemented in the Geant4 *medical_linac* example replicates a GE Saturne 43 linear accelerator. The detailed description of the accelerator and the phantom are provided in the EURADOS Report. The experimental data were acquired with the GE Saturne 43 accelerator at Laboratoire National Henri Becquerel (CEA, LIST, LNE LNHB).

#### 3.3.1 Simulation set‐up

The simulation set‐up models the GE Saturne 43 accelerator, with a nominal energy of 12 MeV, a source‐skin distance (SSD) of 90 cm and a field size of 10 × 10 ${\text{cm}}^{2}$ using a liquid water phantom as detector. The energy and dimension of the electron source, and the field size can be easily adjusted via a macro file.

We modeled the electron source as a uniform disc with a 2 mm diameter, normally incident on the target, with a Gaussian energy distribution. Tests with different source sizes and various full width at half maximum (FWHM) were performed to ensure sufficient beam uniformity while minimizing edge effects, which is crucial for accurate dose calculations.^47^, ^48^

We simulated energies varying between 11.4 and 12.2 MeV with a step of 0.2 MeV. The energy of the primary electrons impacts the depth dose distribution^49^ and the optimized energy of 11.6 MeV was determined by comparing percent depth dose (PDD) and transverse normalized profile curves simulated using *Option4* with measured data. For the FWHM values, we considered that it has a minimal influence on the central‐axis relative depth‐dose for a 10 × 10 ${\text{cm}}^{2}$ field size, so we considered its impact on the dose profile. The FWHM was set to 0.3 MeV, representing a relatively narrow energy spread. A wider distribution, with a larger spread in electron energies, could affect the precision of the simulation and the consistency of the results.^47^, ^48^, ^49^, ^50^

**FIGURE 3:**
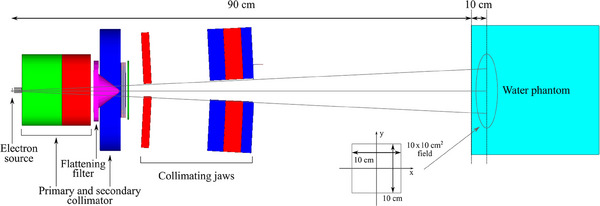
Schematic view of the simulation set‐up of the *MV x‐ray radiotherapy* test, which is released in the Geant4 *medical_linac* advanced example and based on the geometry of the Saturne 43 General Electric linear accelerator. The 10 × 10‐cm^2^ field size is defined at 100 cm from the source, 10 cm below the phantom surface. The diagram is not to scale.

The adjustable collimating jaws are set in order to obtain a field of 10 × 10 ${\text{cm}}^{2}$ at a distance of 100 cm from the electron source, as shown in Figure 3. A 25.5 × 25.5 × 25.5‐${\text{cm}}^{3}$ water phantom is positioned at 90 cm from the electron source, that is, the surface of the phantom lies at 90 cm from the proximal surface of the target. A Geant4 cubic scoring mesh with the same size of the phantom and voxels of 5‐mm width is used to score the dose. The voxel centers are aligned with the experimental data points.

The production cuts are set to a maximum of 20% of the minimum thickness of each beamline component and, in the phantom, 20% of the voxel size. A detailed list of the cuts used is reported in Table 4.

**TABLE 4:** Production cuts used in the components of the beamline.

Beamline component	Thickness (mm)	Cut (mm)
Target (tungsten)	4	1.0
Primary collimator	79 + 57.5	10.0
Flattening filter	min 7.5, max 46.8	1.5
Secondary collimator	40.5	10.0
Phantom	5 (voxel side)	0.1

The γ index test^51^ was used to evaluate the discrepancies between the experimental data and the simulated data (3%/3 mm criterion was chosen).

The test has been performed using the EM constructors *Opt3*, *Opt4*, *Livermore*, and *Penelope* to assess the impact of the different EM physics constructors on the dose calculation.

The statistical uncertainties have been calculated with the history‐by‐history method^52^ and the photons are produced from independent primary electrons, with neither biasing nor recycling.

#### 3.3.2 Results and discussion

The reported results are obtained using Geant4 11.1. 2 × 10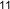 primary events are set to enable the simulation of the dose distribution in a water phantom positioned at a distance of 90 cm from the linear accelerator source with an uncertainty lower than 1%.

**FIGURE 4:**
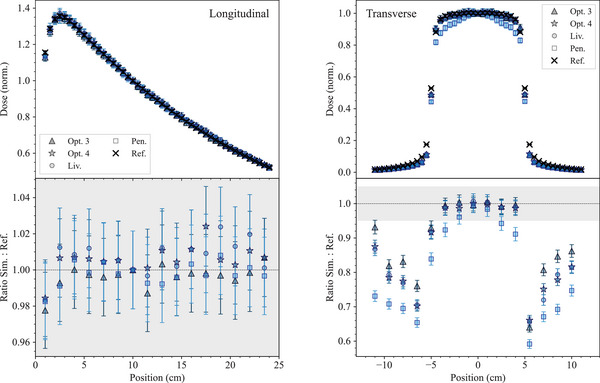
Results of the *MV x‐ray radiotherapy* test obtained with Geant4.11.1. Top left: longitudinal dose distribution in the water phantom. The curves are normalized to the dose value at a depth of 10 cm. Top right: transverse dose distribution at 10‐cm depth in the water phantom. The curves are normalized at the center; the uncertainties of the simulation results are within symbols. Bottom: ratios of simulated and experimental data for the longitudinal (left) and transverse (right) profile. The shadowed area represents an agreement within 5% with the reference data. Experimental data from Caccia et al.^46^

Figure 4 shows the normalized dose profiles along the longitudinal axis, and the transverse dose profiles at a depth of 10 cm in the water phantom, obtained with all the EM constructors. The normalization is performed to the dose value at a depth of 10 cm for the longitudinal profile, and at the center for the transverse profile. Also the ratio of simulation results and reference data is shown. In the longitudinal profile, a 95% confidence level agreement is observed for all the EM physics constructors. For the transverse dose distribution (10 cm below the water phantom surface), simulated and experimental data are in agreement within 3% in‐field for the EM constructors *Opt3*, *Opt4*, and *Livermore*, while larger differences are observed for the *Penelope* EM constructor, which are discussed more in detail later in this section. Discrepancies at the edge of the field area are attributed to the high dose gradient of this region where the uncertainty in the position is critical.

**FIGURE 5:**
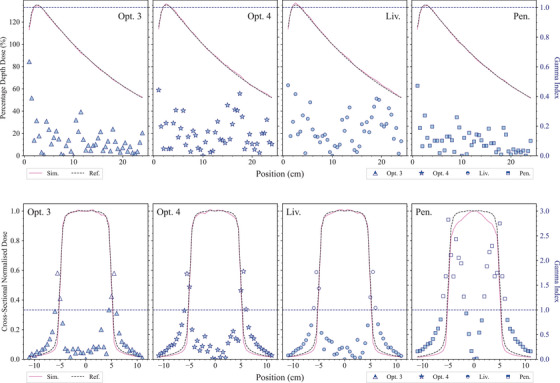
Results of the γ index test for all the tested EM constructors (*Opt3*, *Opt4*, *Livermore*, and *Penelope*). Top row: longitudinal profiles. Bottom row: transverse profiles. Dotted line signifies a γ index of 1.

To complete the analysis, the γ index test was performed and the results are shown in Figure 5. The comparison results show that 100% of the points of the longitudinal dose profile have a γ index (3%/3 mm) below 1, for all the EM physics constructors under testing. In the case of the transverse dose profile, about 90% of the data points have a γ index below 1 for *Opt3*, *Opt4*, and *Livermore*. Less agreement (60%) is obtained with the *Penelope* EM constructor.

**FIGURE 6:**
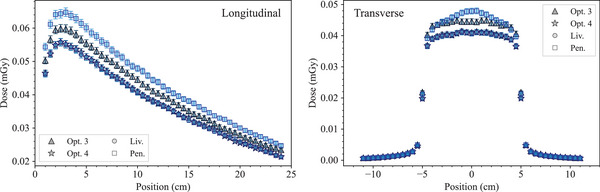
Absolute dose profiles in the water phantom, obtained with $2\times 10^{11}$ histories and the four EM constructors. Left: longitudinal profile. Right: transverse profile at 10 cm of depth. Results obtained with Geant4.11.1.

Figure 6 reports the absolute dose values—that is the cumulative dose due to all the primary histories—along the longitudinal and transverse profiles simulated with the four EM constructors. The differences, with respect to *Opt4*, in the first 10 cm of water phantom for the longitudinal profiles are about 1% for *Livermore*, between 6 and 10% for *Opt3*, and between 15 and 16% for *Penelope*. While for the transverse profiles, the differences in‐field are of 1% for *Livermore*, between 7 and 11% for *Opt3*, and between 6 and 17% for *Penelope*.

**FIGURE 7:**
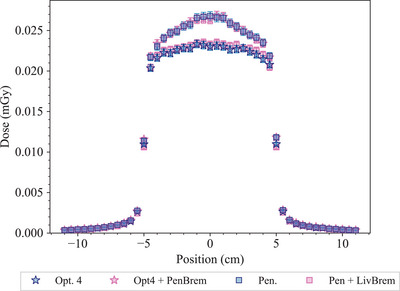
Transverse dose profiles simulated with the full EM constructors *Opt4*, *Penelope*, and the same constructors using a different bremsstrahlung model: *Opt4* with *G4PenelopeBremsstrahlungModel*, and *Penelope* with *G4LivermoreBremsstrahlungModel*. Results obtained with $10^{11}$ histories.

The poor agreement observed in the transverse profile obtained with *Penelope* with the experimental data in‐field has been investigated as follows. In order to isolate the source of the discrepancy, we performed a simulation using the *Opt4* and *Penelope* EM constructors and substituting their bremsstrahlung models. For this test 10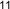 primary histories with 12.0 MeV of energy (the linac nominal energy) have been generated. Figure 7 reports the transverse profiles obtained with *Opt4*, *Opt4* with *G4PenelopeBremsstrahlungModel*, *Penelope*, and *Penelope* with *G4LivermoreBremsstrahlungModel*. The results show that the origin of the discrepancy is related to the bremsstrahlung model. Based on these results, we will further investigate the bremsstrahlung models available in Geant4 to understand more in detail the observed differences.

### 3.4 Electron FLASH radiotherapy test

Among the most innovative radiotherapic techniques recently developed, FLASH radiotherapy, consisting of using ultra‐high dose rate (UHDR) particle beams (40 Gy/s), is attracting an increasing interest in the medical physics community.

Preclinical studies have shown that the use of UHDR beams may substantially improve normal tissue sparing (so‐called FLASH effect) while maintaining the same tumor control probability (TCP) compared to conventional dose‐rate radiotherapy.^53^, ^54^ A clinical translation of FLASH radiotherapy certainly requires the establishment of new protocols and guidelines for the beam delivery, and absolute and reference dosimetry.^55^

For this reason, an extensive effort is currently dedicated to accurately model with Monte Carlo simulations the accelerator machines and the generated radiation field (from 1 cm up to 12 cm diameter), to be able to predict the beam quality and parameters at the irradiation point. In this context, within the framework of a consolidated collaboration between the CPFR (Centro Pisano Flash Radiotherapy) in Pisa and the INFN Catania Division, Italy, a Triode Electron Gun Equipped ElectronFlash Linac, manufactured by Sordina Iort Technologies S.p.A., was simulated in Geant4. The Linac, installed at the CPFR,^56^, ^57^ is able to accelerate 7 and 9 MeV pulsed electron beams at UHDR regime, and the device has been recently simulated in Geant4.

The test, presented here, is based on the *eFLASH_radiotherapy* Geant4 advanced example. There are some differences between the two in terms of beamline modeling as the real Linac geometry is protected by a nondisclosure agreement with third parties. The simulation calculates and benchmarks the lateral dose distribution obtained at the reference position, namely 13 mm water depth, and the depth‐dose distribution in water obtained at the exit of the applicator, with experimental data acquired during the commissioning phase of the EF linac in Pisa and reported in DiMartino et al.^57^

#### 3.4.1 Simulation set‐up

**FIGURE 8:**
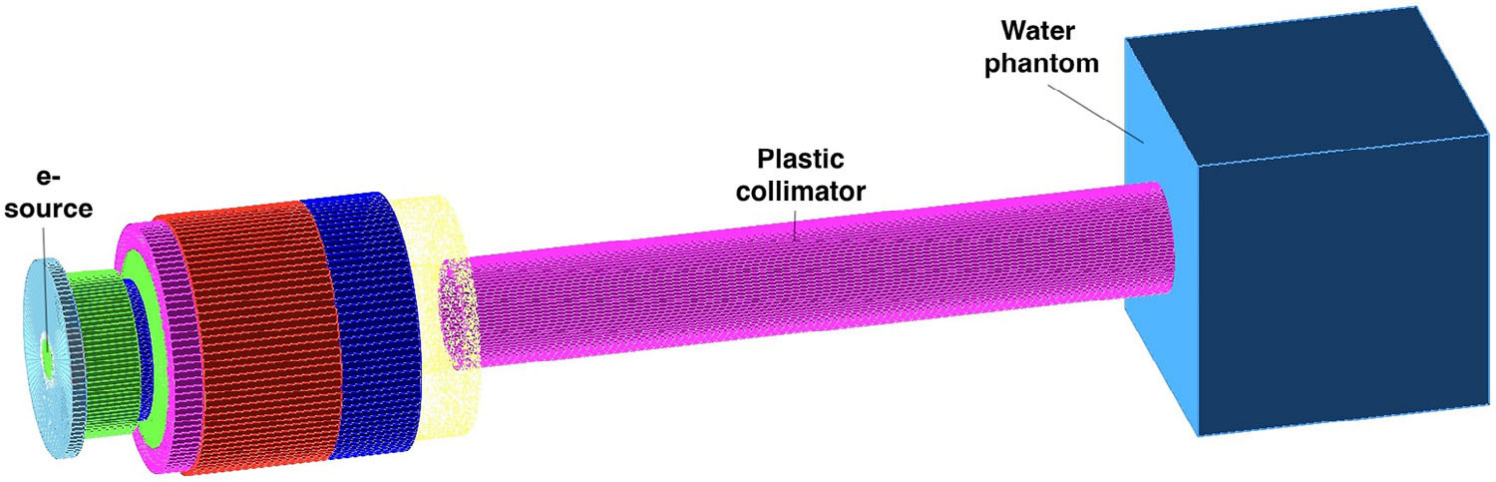
Schematic view of the simulation set‐up of the *electron FLASH radiotherapy* test. The different components are indicated in the figure: the position of the electron source, the plastic shaping applicator, and the water phantom where the dose distributions are computed.

The simulation set‐up is shown in Figure 8, it accurately models the geometrical configuration experimentally used in the preclinical research performed at the CPFR.

The primary radiation field is modeled as a 2.5 × 2.5 ${\text{mm}}^{2}$ electron source with an energy spectrum peaked at 9 MeV, which realistically reproduces the energy spectrum of electrons accelerated by the EF LINAC. The realistic energy spectrum was provided for the G4‐Med test by the manufacturer (SIT Sordina) to assure a direct comparison of the simulations with the experimental data. For copyright reasons, a sightly modified version of the LINAC geometry and source energy spectrum is included in the released Geant4 *eFLASH_radiotherapy* advanced example.

A water phantom is added downstream the accelerator at a distance of 73 cm. A plexiglass 73 cm long cylindric hollow applicator with 10 cm internal diameter is added to shape the field size and obtain a uniform dose distribution with a flat lateral profile.^57^

The depth‐dose distribution along the water phantom is calculated using a scoring mesh with size 60 × 2 × 2$\;{\text{mm}}^{3}$. The mesh is divided in 60 voxels along the x‐axis (the voxel size is then 1 × 2 × 2 ${\text{mm}}^{3}$). The vertical transversal profile is retrieved at 13 mm depth in the water phantom, which corresponds to the maximum expected dose in the depth‐dose distribution.^57^ To calculate the transversal dose profile, a second scoring mesh has been added with size 1 × 120 × 1 ${\text{mm}}^{3}$. This has been divided in 60 voxels along the y‐axis (the voxel size is then 1 × 2 × 1 ${\text{mm}}^{3}$). The EM constructors *Opt3, Opt4, Livermore*, and *Penelope* are tested. A global production cut of 0.1 mm is used. 10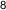 histories are set to obtain a statistical uncertainty lower than 1%. The statistical uncertainty was calculated by executing 10 separate simulations with the same number of events and different random seed.

#### 3.4.2 Results and discussion

**FIGURE 9:**
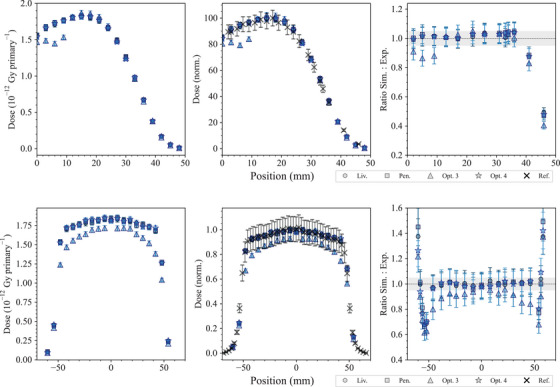
Results of the *electron FLASH radiotherapy* test. Top: longitudinal dose distribution in the water phantom. When not visible, the error bars are within the symbols. Bottom: transverse dose distribution at 13.5 mm depth in the water phantom. The curves are shown in absolute dose normalized to the number of primary particles (left) and normalized to the maximum dose calculated with *Opt4* (at 17 mm depth in the water phantom for the longitudinal distribution) (right). The plots in the last column show the ratio of the Geant4 simulations and reference data reported in DiMartino et al.^57^ The shadowed area represents an agreement within 5%. Results obtained with Geant4 11.1.

Figure 9 shows the longitudinal and the transverse dose distributions in the water phantom obtained with the *electron FLASH radiotherapy* test. The results concerning the longitudinal dose distribution show an agreement between the simulation results obtained with *Opt4*, *Livermore*, and *Penelope* within 2σ, from the surface of the phantom down to approximately 3.5 cm depth. After that the agreement deteriorates; to note, at these distances, the dose is close to zero. *Opt3* shows significant differences, up to 20%, from the surface of the water phantom to 1 cm depth. Then, the agreement is similar to the other EM physics constructors when compared to the experimental data.

**FIGURE 10:**
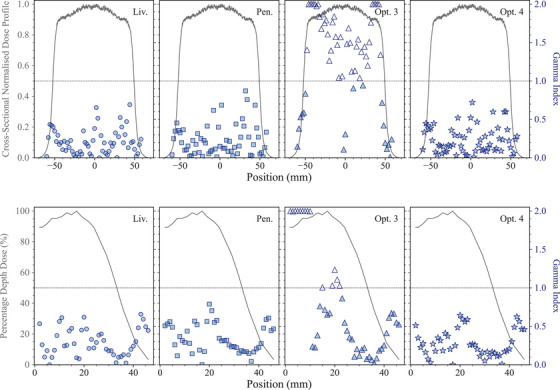
γ index test results for the transversal (top) and longitudinal (bottom) dose profiles obtained with *Livermore*, *Penelope*, *Opt3*, and *Opt4*.

In terms of lateral dose distributions, it can be noted that *Opt4*, *Livermore*, and *Penelope* provide a similar agreement with respect to the reference data, in‐field. Out‐of‐field, there are larger differences, up to 50%, which are attributed to the high‐dose gradient of this region where the uncertainty in the position can be relevant. *Opt3* underestimates the dose in‐field when compared to the other EM physics constructors. However, in order to evaluate more accurately the difference between the simulated results and the experimental data, especially in the regions of the dose profile with a high gradient, the γ index test was performed for both the transversal and longitudinal dose profiles for all the Geant4 EM constructors under study, using a criterion of 4%/2 mm. A dose discrepancy (DD) of 4% and a distance‐to‐agreement (DTA) of 2% were chosen to take into account of the high dose uncertainty and high spatial resolution of the radiochromic films, respectively. As shown in Figure 10, all the EM constructors satisfy the condition of γ<1 apart from *Opt3*.

**FIGURE 11:**
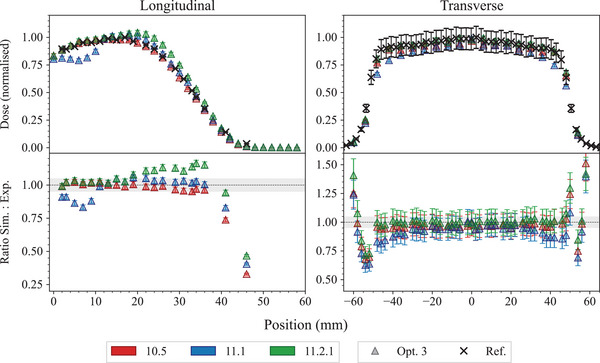
Results of the *electron FLASH radiotherapy* test, obtained with Geant4 11.2.1 and *Opt3* EM constructor. The RangeFactor is 0.04 and the step limitation option is *UseDistanceToBoundary* (only two parameters that have been changed between Geant4 11.1 and 11.2.1 in *Opt3*). The shadowed area represents an agreement within 5%. When not visible, the error bars are within the symbols. Reference data from DiMartino et al.^57^

The large differences found in the depth‐dose and transverse dose profiles when using *Opt3* are due to paramaters of the multiple scattering model. The *RangeFactor* in Geant4 11.1 is 0.03 and the step limitation option is *UseSafetyPlus* (see Section 3.1). Based on the results of this test, a Geant4 patch, 11.2.1, has been released where both parameters have been reverted to the Geant4 10.5 variant. Figure 11 shows the new dose distributions obtained with reverting the multiple scattering parameters of *Opt3* in Geant4 11.2.1. It is possible to observe that there is a better agreement between Geant4 11.2.1 and the reference data when compared to Geant4 11.1. Still, there are some discrepancies in the longitudinal dose distribution, which are under investigation.

### 3.5 Regression testing results

The regression testing performed with the *electron electronic stopping power* test, *photon attenuation* test, *monoenergetic x‐ray internal breast dosimetry* test and *Brachytherapy* test with an 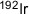 source have reported no differences between Geant4 10.5 and 11.1, and, therefore, are not documented in this report. The reader can refer to the geant‐val website^15^ and to Archer^32^ to access the data analysis.

#### 3.5.1 Electron backscattering test

This test, documented in Dondero et al.^16^ and Arce et al.,^13^ calculates the fraction of electrons backscattered by a circular metallic target in vacuum and the electron backscattering coefficient, η, is defined as follows:

(4) $$\eta =\frac{e_{\text{back}}}{e_{\text{tot}}},$$

where $e_{\text{back}}$ is the number of backscattered electrons and $e_{\text{tot}}$ is the total number of incident electrons. The backscattering coefficient has been calculated with the Geant4 EM contructors *Opt3*, *Opt4*, *SS*, *Livermore*, and *Penelope*. The statistical error affecting the backscattering coefficients is calculated by propagating the uncertainties of the two terms in the fraction, each of which corresponds to the statistical errors of their respective counts.

The simulation application reproduces the irradiation configurations of the Sandia Lab experimental data,^58^, ^59^ used as reference in this work.

In this section, we report the results of the regression testing between Geant4 10.5 and 11.1, considering Be, C, Al, Ti, Mo, Ta, and U targets. The energy of the incident electrons varies from approximately 0.1 keV to 1 MeV. The angle of incidence of the electron beam varies from 0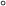 to 75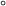. The simulation results are compared to Sandia Lab experimental data.^58^, ^59^ In addition, we report for the first time the benchmarking of the results of this test against a new experimental data set, obtained within the *EXACRAD* Project of the European Space Agency^1^.

**FIGURE 12:**
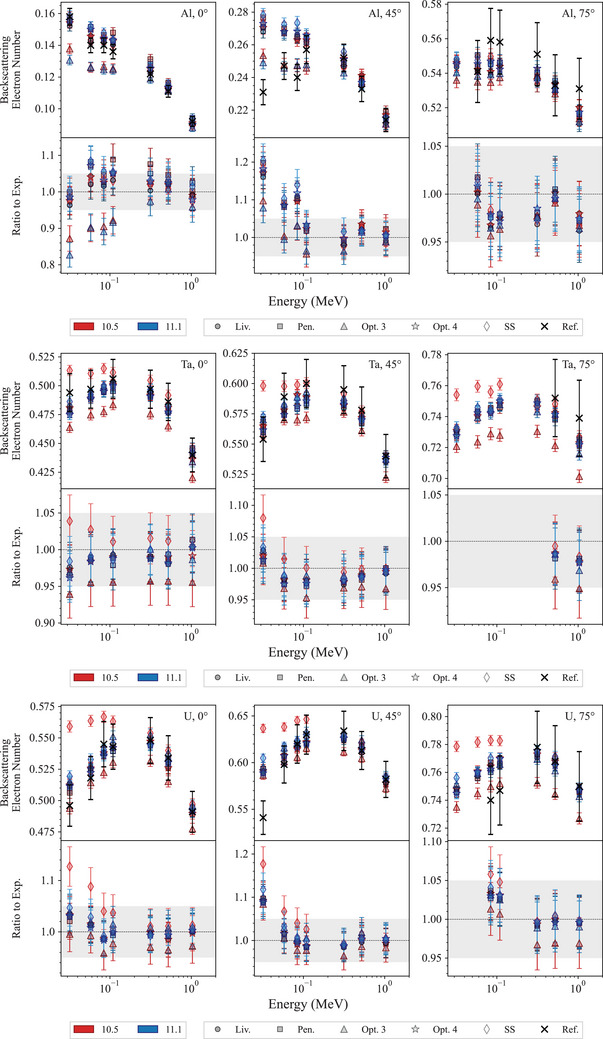
Exemplary electron backscattering coefficients calculated for Al, Ta, and U targets, for different angles of incidence, compared against the Sandia Lab experimental data.^58^, ^59^ The shadowed area represents a 5% agreement. When not visible, the error bars are within the symbols.

Figure 12 shows exemplary results of the regression testing when the Geant4‐calculated backscattering coefficient is compared against the Sandia Lab experimental data.^58^, ^59^

In the case of an aluminum target, when the electron beam is incident normally on the target (0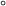 incidence), all the Geant4 EM constructors provide a similar level of agreement with respect to the reference data (within 2σ), with the exception of *Opt3* that tends to underestimate the backscattering electron coefficient, for both Geant4 10.5 and 11.1. In the case of 45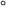 degrees incidence, all EM constructors have an overall agreement within 2σ for energies above 100 keV. For lower energies, *Opt3* provides a better description of the backscattering coefficient when compared to the other EM constructors. When considering a 75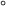 degree incidence, all the simulation results agree with the reference data within 2σ.

When considering the tantalum target, Geant4 11.1 shows an overall better agreement for *Opt3* and *SS* EM constructors, when compared to Geant4 10.5; for this release, all the Geant4 EM constructors show an agreement within 2σ with the reference data.

In the case of an uranium target with a normal incidence direction, all simulation results have an agreement within 2σ with the experimental results apart from the case of the *SS* constructor with Geant4 10.5, when the energy of the incident electrons is below 60 keV. Geant4 11.1 shows a better agreement than Geant4 10.5 with respect to the reference data in the case of the *SS* constructor (within 2σ). When the electron beam is incident with an angle of 45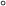, the results obtained with *Opt3*, *Opt4*, *Livermore*, and *Penelope* agree with the reference data within 2σ apart from the case of the lowest electron kinetic energy (32. keV), where the difference is above 8%. In the case of the *SS* constructor, the results agree with the reference data within 2σ when the electron energy is above 60 keV for both Geant4 10.5 and 11.1. For lower energies, the difference is in the range of approximately 6%–18% and 4%–12% for Geant4 10.5 and 11.1, respectively, indicating a clear improvement for Geant4 11.1. In the case of 75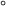 incidence, the agreement with the reference data is within 2σ for all the EM physics constructors apart from the case of the EM constructor *SS*, for Geant4 10.5.

**FIGURE 13:**
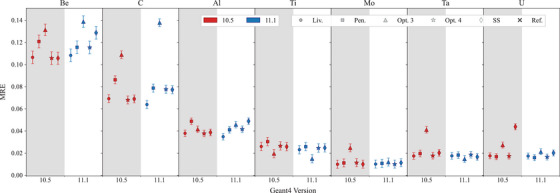
Mean relative error (MRE) calculated for Be, C, Al, Ti, Mo, Ta, and U targets, compared against the Sandia Lab experimental data.^58^, ^59^ The MRE is calculated over the incident angles and electron energies under study. Here the uncertainties are provided as 1σ (see Section 2.1).

Figure 13 reports the MRE for all the targets under study. It can be noted that for targets with lower atomic number (Be, C and Al), *Opt3* and *SS* in Geant4 11.1 tend to provide similar or less overall agreement to the reference data when compared to Geant4 10.5. Instead, for higher atomic numbers (Mo, Ta, and U), Geant4 11.1 shows an overall improvement for *Opt3* and *SS* constructors. The observed differences in terms of backscattering coefficients are due to modifications of the multiple scattering. Provided that the phenomenon of backscattering is more prominent for targets with higher atomic number, the results of this test show an overall improvement of *Opt3* and *SS* in terms of backscattering modeling.

**FIGURE 14:**
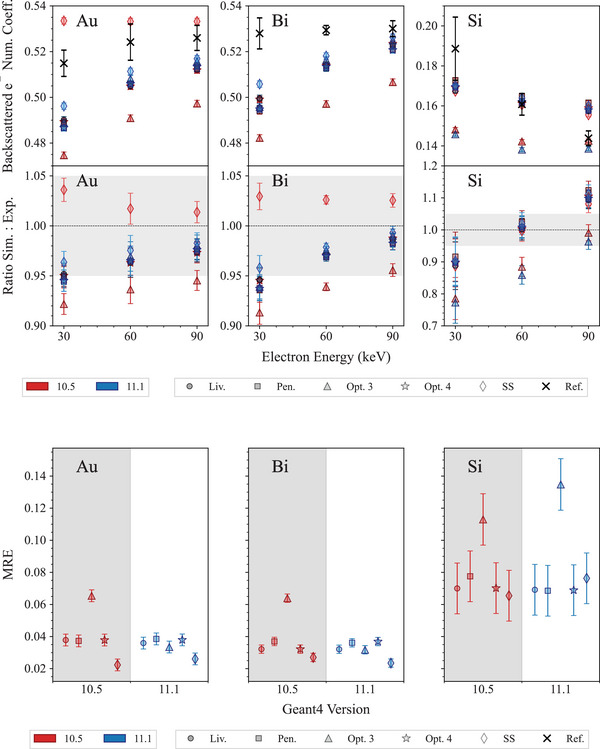
Top: electron backscattering coefficients and ratios to reference data calculated for 30, 60, and 90 keV electrons, incident on Au, Bi, and Si targets. The reference data are experimental measurements of the project Exacrad of the European Space Agency. The shadowed area shows an agreement within 5%. Bottom: mean relative error (MRE), calculated for each EM physics constructor under study. Here the uncertainties are provided as 1σ (see Section 2.1).

Figure 14 shows the results concerning the test calculating the backscattering coefficient for 30, 60, and 90 keV electrons, incident normally on the target, compared against the experimental measurements of the project Exacrad of the European Space Agency.

In the case of an Au target, the *SS* EM constructor provides a different description of the number of backscattered electrons depending on the version of Geant4 (Geant4 10.5 and 11.1 tend to overstimate and understimate the backscattered electron coefficient, respectively), nevertheless the overall agreement with the reference data is within 5%. The results obtained with *Opt4*, *Penelope*, and *Livermore* show an agreement within 6% for both releases of Geant4. *Opt3* shows a better agreement with the reference data in the case of Geant4 11.1 (within 6% against 8% for the case of Geant4 10.5). No release of Geant4 agrees within 2σ with the reference data.

In the case of the Bi target, again the *SS* constructor shows different descriptions of the electron backscattering coefficient depending on the release of Geant4 (Geant4 11.1 underestimating and Geant4 10.5 overestimating η similarly to the Au target). Nevertheless, both releases show similar agreements with the reference data (within 3% for Geant4 10.5 and 4% for Geant4 11.1). Similarly to the case of the Au target, *Opt3* shows a better agreement with the reference data in the case of Geant4 11.1 (within 6% against 9% for the case of Geant4 10.5). No release of Geant4 agrees within 2σ with the reference data apart at energy eqaul to 90 keV for *Opt4*, *Penelope*, and *Livermore*.

In the case of the silicon target, all EM constructors show an agreement within 10% with the reference data, apart from *Opt3* that shows worse results for both Geant4 10.5 and 11.1 (agreement within approximately 25%).

In summary, this test shows an improvement in electron backscattering coefficient calculation for Geant4 11.1 in the case of *Opt3*, for gold and bismuth targets (high‐Z targets). This improvement is ascribed to the revision of the multiple scattering parameters for this EM constructor (see Table 3). *SS* constructor provides different calculations of electron backscattering coefficients for the high‐Z targets but the overall agreement is similar. *Livermore*, *Opt4*, and *Penelope* exhibit a similar overall agreement with the reference data, attributed to their adoption of the same multiple scattering model and parameters (see Tables 2 and 3). For these constructors, there is no significant difference between Geant4 10.5 and 11.1.

#### 3.5.2 13 MeV electron forward scatter from foils

This test, documented in detail in Arce et al.,^13^ is an experimental benchmark of the scatter of electrons with energy equal to 13 MeV, for a comprehensive set of scattering materials (Be, C, Al, Ti, Cu, Ta, Au), for thicknesses that result in a characteristic angle (or root mean square scattering angle) of 2
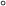
–8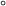.^17^, ^60^, ^61^ The test is of particular interest for the benchmarking of Geant4 for electron therapy when using medical linear accelerators with thin targets intended to minimize electron energy loss and the generation of x‐rays.

The 13 MeV electron beam has an experimental energy spread that is sufficiently small to consider it mono‐energetic. The beam has a spot size of 0.1 cm FWHM and is normally incident on the exit window, a scattering foil of different thicknesses and compositions, a monitor chamber, and mylar slabs on either side of a region filled with helium. The scoring plane is perpendicular to the beam axis and is located 1.182 m from the exit window. The fluence of electrons is scored in radial spatial bins of 1 mm width.

**FIGURE 15:**
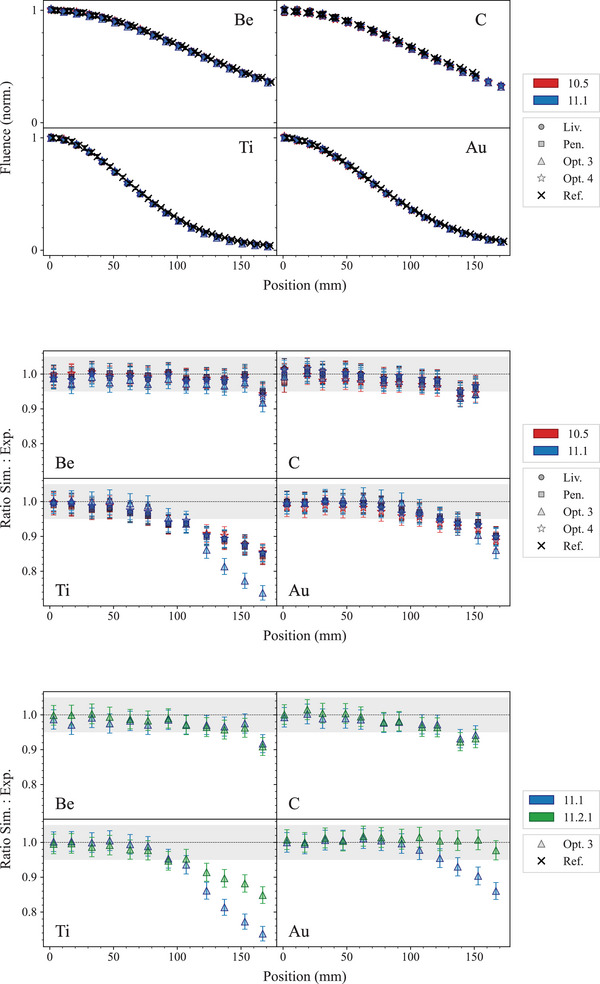
Top: fluence of forward‐scattered electrons obtained with a 13‐MeV electron beam incident on the following foils: 0.926 g/${\text{cm}}^{2}$ Be, 0.546 g/${\text{cm}}^{2}$ C, 0.14 g/${\text{cm}}^{2}$ Al, 0.0910 g/${\text{cm}}^{2}$ Ti, 0.0864‐g/${\text{cm}}^{2}$ Cu, 0.443 g/${\text{cm}}^{2}$ Ta, and 0.0312 g/${\text{cm}}^{2}$ Au. When not visible, the error bars are within the symbols. Middle: ratio of the fluences of the forward‐scattered electrons, plotted against the lateral position, for the different foils, for Geant4 10.5 and 11.1. Bottom: ratio of the fluences of the forward‐scattered electrons, for *Opt3* with Geant4 versions 11.1 and 11.2.1. The shadowed area represents an agreement of 5%.

Figure 15 shows the results of the test. The statistical uncertainty of the simulation results was calculated with the batch method, that is, splitting the simulations in several jobs with different random seeds and retrieving the mean and standard error. This is negligible when compared to the uncertainty affecting the experimental data. Therefore, the uncertainty in the results is the published experimental uncertainty.

The agreement between Geant4 simulations and experimental data is generally within 2σ for all the EM constructors under study, when considering the Be and C targets, for both Geant4 10.5 and 11.1. All EM constructors provide an agreement within 2σ up to 80 and 100 mm for the Ti and Au targets, respectively, for both releases of Geant4. For larger distances, the agreement deteriorates down to 16% for all EM constructors apart from *Opt3* in the case of Geant4 11.1, especially for the Ti targets, where differences up to 28% are observed. The bottom plot of Figure 15 shows the ratio of the simulation and experimental data for *Opt3* and Geant4 11.2.1 and it is possible to observe that the agreement with experimental data improves again for this version of Geant4, due to the changes in the multiple scattering parameters of the Geant4 EM constructor *Opt3*. These results are consistent with the findings of the *electron FLASH radiotherapy* test (see Section 3.4).

#### 3.5.3 Bremsstrahlung from thick targets

This test focuses on the capability of Geant4 to model bremsstrahlung radiation produced by thick targets of Al and Pb. Here, a brief description of the test is provided. The reader can refer to Arce et al.^13^ for more details.

A mono‐energetic electron beam with a diameter of 0.35 cm and energy 15.18 MeV is normally incident with constant fluence on a titanium exit window, followed by a silicon transmission current monitor, and, finally, a pure target encased in a steel target chamber. The photon fluence is scored on the surface of several concentric spherical rings in a sphere of 1 m radius centered at the intersection of the beam axis with the upstream face of the target. A global production cut of 0.01 mm is adopted. The simulation results are compared to experiment (data documented in Faddegon et al.^62^, ^63^).

The results of this test were first reported in Arce et al.^13^ for Geant4 10.5. Nevertheless, in that release, Geant4 did not propagate the statistical weight correctly and the results shown used a local version of Geant4 10.5 where this problem was fixed. The problem was then fixed in the kernel of Geant4 in later releases of the Monte Carlo Toolkit and, here, we report the new results obtained with the test for Geant4 11.1.

**FIGURE 16:**
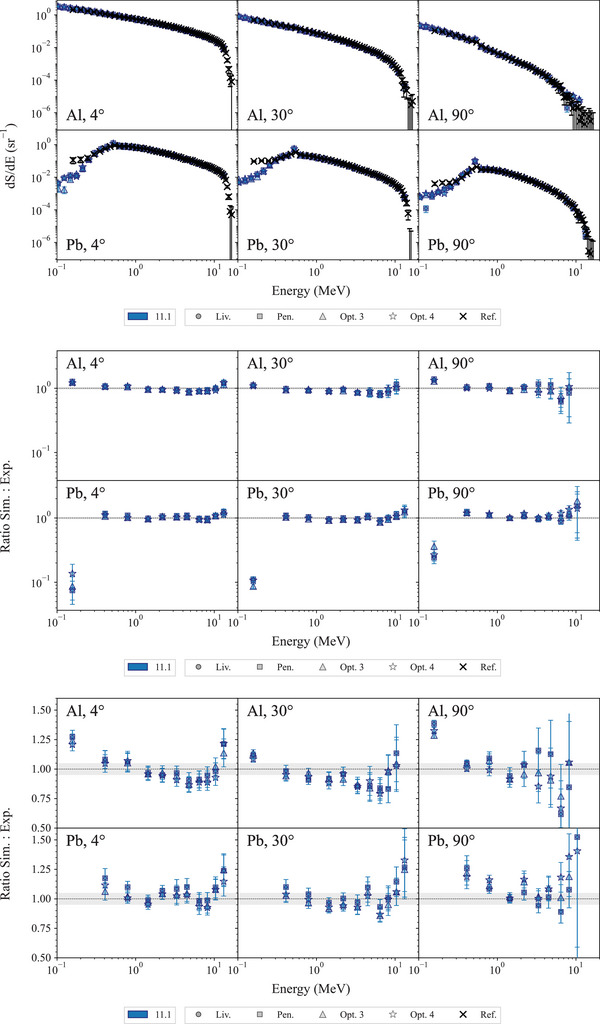
Top: Bremsstrahlung spectra obtained with a 15.18 MeV electron beam incident on targets of Al and Pb. Middle: ratio of the simulation results to the experimental reference data in log scale. Bottom: ratio of the simulation results to the experimental reference data on a linear scale. *Note*: Only every *fifth* data point is shown on the ratio plots for clarity and points outside the axis limits are not visible in the linear scaled ratio plot but are shown in the log scaled plot. The shadowed area represents an agreement within 5%.

The top plot of Figure 16 shows the bremsstrahlung spectra obtained with Geant4 11.1. The middle plot shows the ratios of the simulation results to the reference experimental measurements in log scale, while the bottom plot shows the ratio in linear scale for photon energies above 0.4 MeV.

The statistical uncertainty of the simulation results was calculated with the batch method. Similarly to the electron forward scattering test 3.5.2, this is negligible when compared to the uncertainty affecting the experimental data, therefore the uncertainty affecting the results is the experimental uncertainty.

In the case of both Al and Pb targets, Geant4 seems to underestimate the photon fluence at energies below approximately 0.3 MeV, when compared to the reference data. This discrepancy, observed for the combination of low energy and low fluence, was attributed in the benchmark paper Faddegon et al.^62^ to collimator effects and charged particle contamination that were not included in the uncertainty estimates.

For both targets, apart the low energy and low fluence combination case, the agreement between Geant4 simulation results and reference data is in general within 2σ for all the Geant4 EM constructors.

#### 3.5.4 Fano cavity test

The Fano cavity test is designed to check the accuracy of the condensed history (CH) electron transport when simulating the response of detectors with gaseous cavities. In particular, the different factors involved in the electron transport, that is, the step limitation, effects of energy loss, modeling of multiple Coulomb scattering, are tested using the Fano cavity principle.^64^ This test is recommended by the AAPM Research Committee Task Group 268.^65^

A beam of 1.25 MeV gamma rays is incident normally and uniformly on the flat ends of an ionization chamber, which consists of a cylinder made of 5 mm water (G4_WATER) walls and a 2 mm cavity filled with steam (G4_WATER with a density of 1.0 mg/${\text{cm}}^{3}$).^66^ The direction of the photon beam is along the cylinder axis and there is particle equilibrium in the ionization chamber.

As explained in Arce et al.,^13^ this test requires specific physics modeling conditions, and the physics constructors used in this test (indicated with a 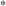 for clarity) differ from the corresponding Geant4 EM physics constructors except the modeling of the electron Coulomb scattering and the corresponding stepping algorithm. Ionization is simulated with the *G4MollerBhabha* model (see the Geant4 Physics Reference Manual^15^ for details). In order to have the same stopping power in the wall and cavity, the density correction term in the electronic stopping power formula is not applied. The Fano Cavity test conditions imply no energy transfer via bremsstrahlung radiation. Therefore this process is not registered.

After each Compton interaction, the scattered photon is reset to its initial state, energy, and direction. Consequently, interaction sites are uniformly distributed within the wall material, obtaining particle equilibrium. With this set‐up, the ratio of the dose deposited divided by the beam energy fluence should be equal to the mass‐energy transfer coefficient of the wall material. The EM physics constructors under study are *Opt3* 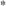 and *Opt4* 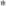. *Livermore* and *Penelope* are not considered as they have the same multiple scattering model of *Opt4*.

**FIGURE 17:**
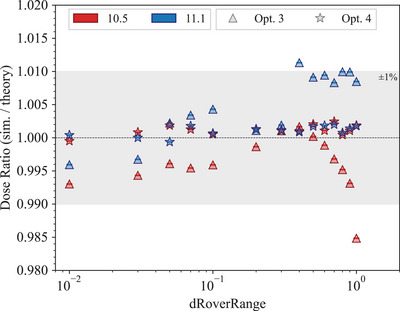
Ratio of simulated dose and the theoretical value plotted against the *dRoverRange* parameter. The shadowed area represents a 1% agreement with the unity.

Figure 17 shows the dose ratio calculated with the simulation and the theoretical value, varying the *dRoverRange*, which is a parameter of the Geant4 multiple scattering. While the results obtained with *Opt4* 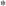 do not change from Geant4 10.5 and 11.1, those with *Opt3* 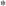 vary, but, still, the agreement with unity is within 1%.

## 4 NUCLEAR FRAGMENTATION CROSS SECTIONS BENCHMARKING TESTS

Three independent tests are included in the G4‐Med benchmarking suite to evaluate the total hadronic inelastic cross section, which is described with the Glauber–Gribov model in Geant4,^33^ and three different nuclear fragmentation models available in Geant4, the Binary Light Ion Cascade (BIC), the Quantum Molecular Dynamics (QMD), and the Liege Cascade (INCL) models,^13^ describing the final state of the fragmentation process.

The BIC models nuclear fragmentation in the energy range between 0 and 6 GeV/u, by describing the interaction between the projectile and the participating nucleons of the target nucleus.^13^ The residual nucleus is in pre‐equilibrium state and is de‐excited to the equilibrium state using the precompound model Geant4.^33^ The final de‐excitation is performed by the Geant4 de‐excitation module. It invokes the Fermi Break‐Up, neutron and light ion evaporation, fission for heavy fragment emission, GEM model for light fragment emission, and photon evaporation to model the nuclear gamma cascade Geant4.^33^ A significant change between Geant4 10.5 and 11.1 is in the modeling of the de‐excitation channels in the Fermi Break‐Up model. Many more reaction channels are considered in Geant4 11.1 (from 991 to 5421 reactions).^67^

The QMD models ion nuclear fragmentation between 100 MeV/u and 10 GeV/u. Below 100 MeV/u, the BIC is used. The QMD describes the interaction between the projectile and all the nucleons of the target nucleus.^13^

The INCL models fragmentation and all the subsequent processes, including nuclear de‐excitation, in the energy range between 0 and 3 GeV/u. The target nucleons are treated as a free Fermi gas in a static potential well, whereas the projectile is modeled without Fermi motion.^13^

Above the limit of applicability of the BIC, QMD, and INCL, the Fritiof parton string model Geant4^33^ is adopted, nevertheless, it is not tested in this work as the energy range is too high for bio‐medical applications.

### 4.1 Regression testing results

#### 4.1.1 Test of nucleus–nucleus hadronic inelastic scattering cross sections

This test, described in detail in Arce et al.,^13^ calculates the total cross section of hadron–nucleus and nucleus–nucleus collisions, which is then compared to reference experimental measurements of the Experimental Nuclear Reaction Data (EXFOR) database.^68^ The total inelastic scattering hadronic cross sections are calculated for incident protons and carbon nuclei, for the following reactions: p+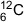, p+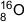, p+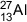, p+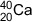, 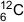
+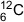, and 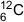
+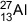. In Geant4, the total inelastic scattering hadronic cross section is based on the Glauber representation with the Gribov screening correction on inelastic screening^69^, ^70^, ^71^, ^72^ and it is used in all the Geant4 hadronic physics constructors.

**FIGURE 18:**
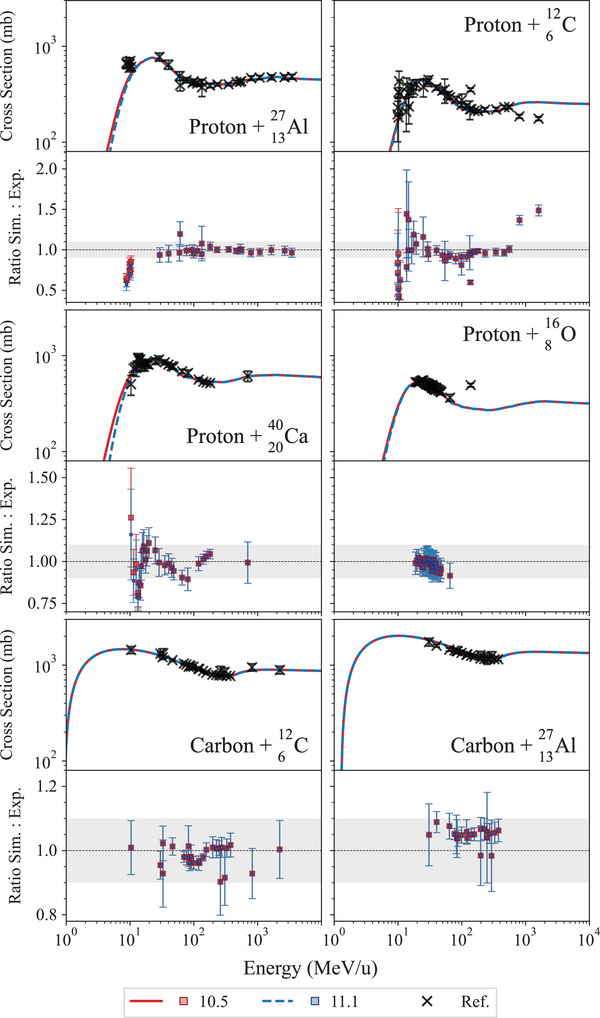
Total inelastic cross sections for the nuclear reactions under study. The Geant4 cross sections, obtained with QGSP_BIC_EMY and represented with red and blue lines for Geant4 10.5 and 11.1, respectively, do not have any statistical uncertainty because they are model‐based calculation. The shadowed area represents an agreement within 10%.

Figure 18 shows the results of the regression testing between Geant4 10.5 and 11.1. The total inelastic cross sections is plotted as a function of the kinetic energy of the projectile and is compared to the experimental data of the EXFOR database. The cross sections do not have any statistical uncertainty because they are model‐based calculation. Both releases of Geant4 provide the same level of agreement with the reference data, for both incident protons and carbon ions. In the case of incident protons, there is an overall agreement within 2σ, apart from some isolated points. In the case of incident carbon ions, the agreement is within 2σ when the target is 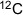, while in the case of the reaction 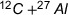, Geant4 consistently overestimates the cross section of about 5%–10%, when compared to the reference data for both releases of Geant4.

**FIGURE 19:**
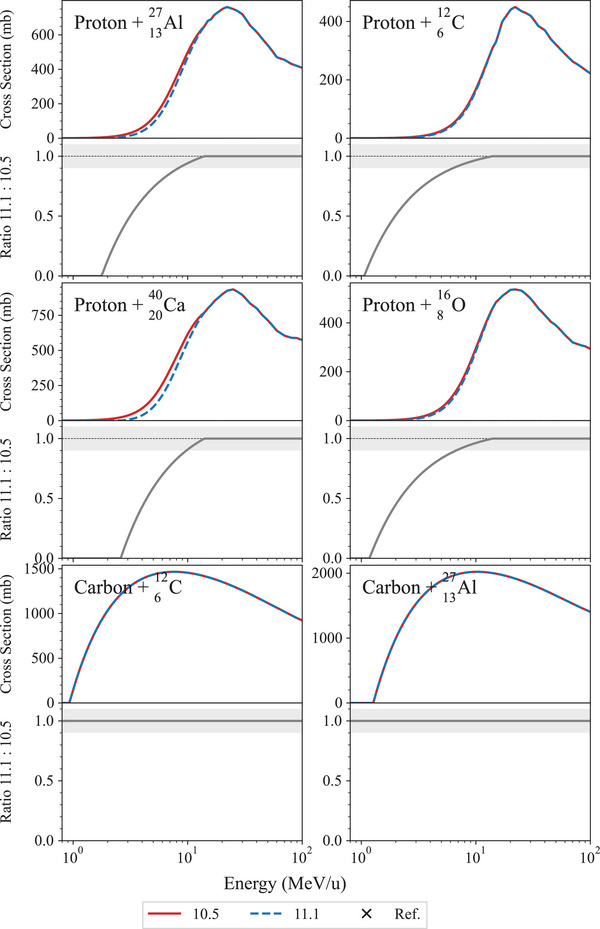
Total inelastic cross section, obtained with QGSP_BIC_EMY and represented with red and blue lines for Geant4 10.5 and 11.1, zoomed in the energy range between 1 and 100 MeV/u, together with the ratios (represented with gray continuous lines) of the cross sections calculated with Geant4 11.1 and 10.5.

Figure 19 shows the total inelastic cross section in the energy range between 1 and 100 MeV/u. It is possible to observe that for incident protons, the cross section is lower up to 10 MeV in the case of Geant4 11.1, while there is no difference for incident carbon nuclei. The difference between Geant4 10.5 and 11.1 proton cross sections is due to the change of Coulomb barrier parameterization in the cross section computation. In 10.5, an empirical parameterization^1^ was used. In version 11.1, hadronic cross sections are obtained from tabulation of ParticleHP evaluated data based on the best known experimental data and at higher energies on the Glauber–Gribov theory.^3^ The approach of Geant4 11.1 is, therefore, deemed more accurate.

#### 4.1.2 62 MeV/u fragmentation test

This test, described in detail in Arce et al.,^13^ calculates the double‐differential cross sections of light fragment production of 62 MeV/u 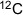 ions incident on a thin natural carbon target. The secondary fragments under investigation are 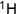, 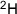, 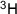, 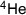, 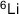, 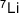, 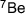, 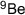, 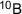, and 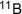. The calculated cross sections are compared to experimental measurements reported in De Napoli et al.^73^ Simulations are executed modeling the nuclear fragmentation with the Binary Light Ions Cascade (BIC)^74^ and the Liege Intranuclear Cascade (INCL).^75^, ^76^ The QMD^77^ is not subject of the test, as, below 100 MeV/u, its Geant4 physics constructor (*G4IonQMDPhysics*) switches to the BIC.^13^

**FIGURE 20:**
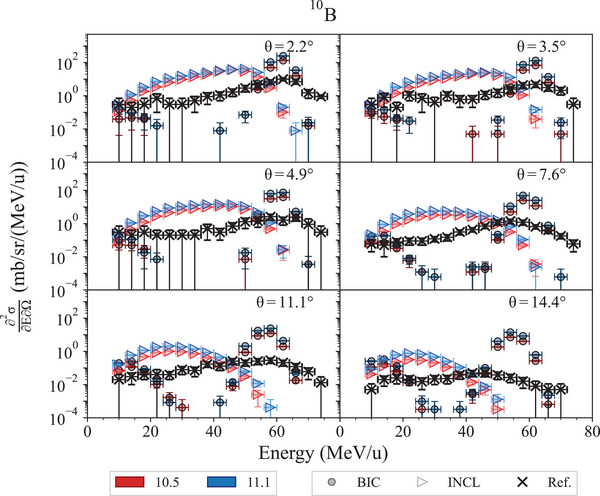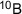 production double‐differential cross sections, obtained with 62 MeV/u 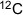 ions incident on a thin natural carbon target, calculated with Geant4 10.5 and 11.1. The simulation uncertainty is calculated, assuming a Poisson distribution, proportional to the square root of the number of events in each bin. Reference experimental measurements from De Napoli et al.^73^

**FIGURE 21:**
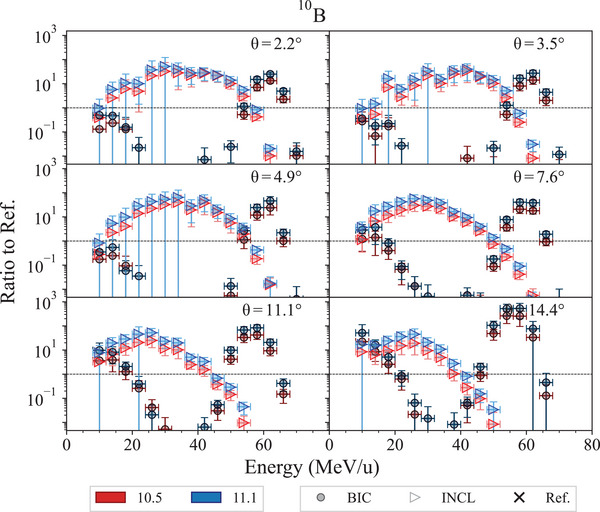
Ratio of the simulation and reference data for 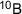 production double‐differential cross sections, obtained with 62 MeV/u 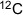 ions incident on a thin natural carbon target. The shadowed area represents a 10% agreement. Reference experimental measurements from De Napoli et al.^73^

The regression testing shows that the cross section of production of 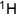 isotopes does not change between Geant4 10.5 and 11.1, while there are some differences for 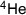, 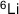, 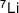, 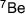, 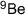, 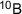, and 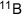, for both INCL and BIC fragmentation models. Nevertheless, the overall agreement against experimental data does not change between Geant4 10.5 and 11.1. Figures 20 and 21 show as exemplary case the double differential cross section of production of 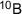 and its ratio against the reference data, respectively. In this case, the differential double cross section is slightly higher in Geant4 11.1 than in Geant4 10.5, but the overall agreement with the reference data remains the same. Specifically, BIC continues to show a doubly peaked structure due to the de‐excitation of the projectile and target remnants, while underestimating the mid‐rapidity region. INCL, on the other hand, continues to overestimate production in that region, showing a single distribution. Possible explanations for these behaviors could include the fact that the BIC approach is based on a time‐invariant optical potential, which leads to an underestimation of neck fragmentation events, whereas INCL uses a complete‐fusion approach.^13^

#### 4.1.3 300 MeV/u ion charge‐changing cross section test

This test, described in detail in Arce et al.,^13^ benchmarks the partial and total charge‐changing cross sections of 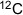 ions with energy 300 MeV/u as simulated by Geant4 against experimental data published in Toshito et al.,^23^ obtained with an emulsion plate in the NIRS P152 experiment.

In this simulation, 300 MeV/u carbon ions are incident on a water (G4_WATER) phantom with a 10 m side. All electromagnetic processes and decay physics are switched off and only hadron and ion transport processes are modeled. Projectile‐like fragments are then identified as they travel in the forward direction (momentum along the initiated direction > 600 MeV/c/u) and retrieved. Partial charge‐changing cross sections are then calculated for B, Be, and Li isotopes. The statistical uncertainty affecting the simulation results is calculated as follows. The $\frac{\sqrt{N}}{N}$ is used to estimate the relative error $\sigma_{SIM}$ of the number N of the generated fragments, assuming a Poisson statistics. Then, the uncertainty of the calculated cross section CS is obtained by multiplying CS with $\sigma_{SIM}$.

**FIGURE 22:**
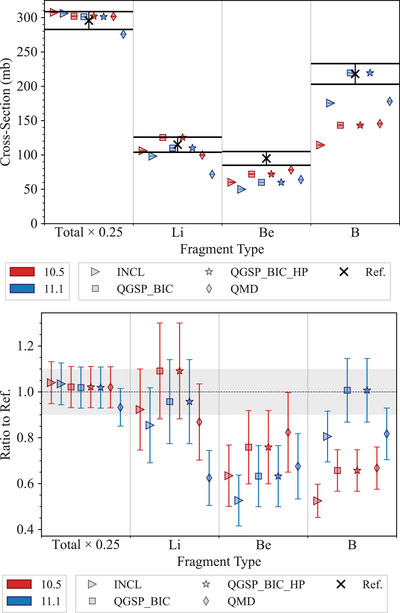
Top: comparison of Geant4 simulation results obtained with Geant4 10.5 and 11.1 against experimental data^23^ for 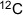 total and partial charge‐changing cross sections. The energy of the incident 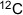 ion beam is 300 MeV/u. The error bars of the simulation results are within the symbols. Bottom: ratio of simulated and reference data for Geant4 10.5 and 11.1. The shadowed area represents an agreement within 10%.

Figure 22 shows the results of the regression testing between Geant4 version 10.5 and 11.1. The bottom plot shows the ratio of the simulation and reference data. The agreement within 2σ is confirmed for the total cross section and Li partial cross section when using Geant4 version 11.1. In the case of the Be partial cross section, Geant4 11.1 provides poorer agreement than 10.5 with respect to the experimental measurements. Depending on the specific fragmentation model, a degradation of the level of agreement of approximately 10%–15% has been found. However, in Geant4 11.1, the calculation of the Boron partial cross section improves significantly, especially for the BIC model. In the case of QMD and INCL, the difference to the reference data decreases to below 20%, while the BIC model provides an agreement with the reference data within 2σ. The increase of B yield is coherent with the increase of the double‐differential cross sections observed for Geant4 11.1 in the test with 62 MeV/u 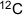 ions incident on the thin natural carbon target (Section 4.1.2).

Overall, in this test, when compared to INCL and QMD, BIC, and BIC_HP provide a better agreement against reference data.

## 5 ELECTROMAGNETIC AND HADRONIC PHYSICS BENCHMARKING TESTS

This section reports the results of the G4‐Med tests where both electromagnetic and hadronic physics processes are objects of the testing. Section 5.1 briefly describes the physics lists of Geant4 benchmarked in this work and summarizes the evolution of the Geant4 hadronic physics from Geant4 10.5 to 11.1, of interest for bio‐medical physics applications. Section 5.2 shows new developments of the *Hadrontherapy* test in terms of experimental microdosimetry. Section 5.3 describes a new test introduced in the G4‐Med benchmarking system for in vivo PET for heavy ions. Finally, Section 5.4 reports significance results of the regression testing of existing tests, documented in Arce et al.^13^ performed with Geant4 10.5 and 11.1.

### 5.1 Evolution of the Geant4 hadronic physics from Geant4 10.5 to 11.1, of interest for bio‐medical applications

The Geant4 physics lists, modeling both electromagnetic and hadronic physics interactions, subject of the benchmarking of Geant4 for hadrontherapy, are *QGSP_BIC_HP*, *QGSP_BIC_AllHP*, *QGSP_BERT_HP*, *QGSP_INCLXX_HP*, and *Shielding*, and they are briefly described in Table 5.

**TABLE 5:** Geant4 physics models of the hadronic physics list used in this report.

Geant4 physics list	QGSP_BIC_HP	*QGSP_BIC_AllHP*	*QGSP_BERT_HP*	*QGSP_INCLXX_HP*	*Shielding*
*EM physics*	*Opt4*		*G4EmStandardPhysics*		
	*G4EmExtraPhysics*				
	Modeling synchrotron radiation and gamma/e‐/e+ nuclear processes				
*Radioactive decay*	*G4RadioactiveDecayPhysics*				
	Modeling of rare decays of hadrons (pion, kaons, etc.) is not relevant for the G4‐Med tests				
*Hadron elastic scattering*	*G4HadronElasticPhysicsHP*			—	—
*Other* hadronic physics processes	*G4HadronPhysics QGSP_BIC_HP* ^a^	*G4HadronPhysics QGSP_BIC_AllHP* ^b^	*G4HadronPhysics QGSP_BERT_HP* ^a^	*G4HadronPhysics INCLXX*	*G4HadronPhysics Shielding* *BERT_HP* ^a^ < 3 GeV, *FTPF_HP* ^a^ > 3 GeV
*Ion elastic physics*	*G4IonElasticPhysics*		—	—	*G4IonElasticPhysics*
*Other ion physics*	*G4IonPhysics* (*G4BinaryLightIonReaction*)	*G4IonPhysicsPHP* (*G4ParticleHPInelastic* < 200 MeV, *G4BinaryLightIonReaction above*)	*G4IonPhysics* (*G4BinaryLightIonReaction*)	*G4IonINCLXXPhysics*	*G4IonQMDPhysics*

^a^ _HP: use of evaluated databases only for neutrons.

^b^ _allHP: use of evaluated databases for *n*, *p*, *d*, *t*, 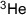, and α particles.

From Geant4 version 10.5 to 11.1, there have been limited changes in the hadronic physics models relevant for bio‐medical applications, in the hadronic inelastic cross sections and physics lists used in this benchmark. Consistency improvements, clean‐up, and optimization of the code have been performed, but without reported relevant impact in the situations of interest in this study, other then the data they use.

New versions of the hadronic data sets are used in Geant4 11.1. First of all, the new G4NDL.4.7, for data‐driven models for neutron transport, is used in the *“_HP”* physics lists. With respect to G4NDL.4.5 (present in Geant4 10.5), the new dataset incorporates new neutron cross sections and final states obtained from the JEFF‐3.3 data library, and includes new materials for the simulation of thermal neutrons. The dataset G4TENDL.1.4, used by the *“_AllHP”* physics lists, was released with Geant4 version 11.0 and uses ENDF/B–VIII.0 and TENDL–2019 libraries (vs. G4TENDL.1.3.2 present in Geant4 version 10.5, which used ENDF/B‐VII.1 and TENDL‐2014 libraries).

With respect to G4PARTICLEXS.1.1 present in Geant4 10.5, the G4PARTICLEXS.4.0 dataset present in Geant4 11.1 was derived using cross section data from G4NDL.4.7 for neutrons with energies below 20 MeV, from G4TENDL.1.4 for protons below 150 MeV and light ions at selected energy ranges, and from new data for gamma‐nuclear cross sections below 130 MeV. This dataset is used in all non‐HP precompiled Physics Lists.^33^ For gamma‐nuclear cross section, the same cross section class *G4PhotoNuclearCrossSection* is used above 130 MeV.

G4ENSDFSTATE.2.3, used in Geant4 11.1 and released with Geant4 10.7, incorporates minor changes in nuclear levels and isomer lifetimes versus G4ENSDFSTATE.2.2 present in version 10.5. After dedicated efforts, G4ENSDFSTATE.2.3 is coherent with G4PhotonEvaporation.5.7 and G4RadioactiveDecay.5.6 datasets.

G4PhotonEvaporation.5.7, released with Geant4 version 10.7, updates data on nuclear levels and transition probabilities versus G4PhotonEvaporation.5.3 in Geant4 10.5. These data are used in the Geant4 nuclear de‐excitation module used in the physics list containing *“BIC,”* *“INCLXX,”* or *“QMD”* in its name, as well as in *“_AllHP”* physics lists.

G4RadioactiveDecay.5.6, released with Geant4 version 10.7, incorporates various updates, additions, and fixes with respect to G4RadioactiveDecay.5.3, present in Geant4 10.5. The radioactive decay module is used by default in the physics list *Shielding* and those including *“_HP”* or *“_AllHP”* in their name.

### 5.2 Hadrontherapy test

The *Hadrontherapy* test is released in Geant4 as an advanced example (called *Hadrontherapy*) since 2003. The test has been included in the G4‐Med benchmarking system since its first release in 2019. It was originally focused on comparing clonogenic cells' Survival Fraction (SF) curves with experimental radiobiological data (see Arce et al.^13^). The regression testing between Geant4 10.5 and 11.1 of the calculation of the SF curve did not report any change between the two releases of Geant4, therefore the results will not be shown here.

An additional functionality has been recently included in the test, focused on the calculation of track‐averaged LET (${\bar{L}}_{T}$) curves and their comparison against experimental microdosimetric data, obtained at the CATANA facility, Laboratori Nazionali del Sud, Istituto Nazionale di Fisica Nucleare in Catania^78^, ^79^ (INFN‐LNS, Italy).

#### 5.2.1 Simulation set‐up

The CATANA protontherapy clinical beamline, installed at LNS‐INFN, is modeled in detail in terms of materials and geometry. A voxelized water phantom is set at the end of the beamline, to replicate a standard water tank, conventionally adopted in a clinical environment, for depth dose curve reconstruction. For this test, the tank is segmented into slices with size 400 × 400 × 0.01 mm^3^.

The proton beam is simulated with a Gaussian energy distribution, centered at a nominal energy of 62.40 MeV and a standard deviation of 0.25 MeV. The beam spot is modeled as circular, following a bivariate Gaussian distribution with a standard deviation of 5 mm. A Gaussian angular distribution is assumed for the beam, with a standard deviation of 0.028º. Proton beam energy modulation is achieved using a plastic modulator wheel, generating a modulation width of 11 mm in water and a maximum proton range of 30 mm in water. Each simulation execution comprises a total of 3.6 × 10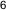 histories. The production cut for secondary electrons, defining the threshold below which secondary particles are not individually generated and tracked, is set equal to 0.1 mm.

The track‐averaged LET, ${\bar{L}}_{T}$, is computed using *QGSP_BIC_HP*, *QGSP_BERT_HP*, and *QGSP_BIC_AllHP*,^33^ and compared against $y_{F}$ values defined in the ICRU Report 36,^80^ derived from experimental microdosimetric spectra acquired at 13 different depths in water. These spectra were obtained using the MicroPlus probe detector^81^ developed at the Centre for Medical and Radiation Physics, University of Wollongong, Australia.^81^

The term *averaged LET* refers to the mean value of the electronic stopping power when considering all particles traversing a specific volume within a radiation field. The track‐averaged LET (${\bar{L}}_{T}$) is calculated as described by Petringa et al.,^24^ with an approach analogous to the method C suggested by Cortés‐Giraldo and Carabe^82^ to avoid biased results:

(5) $${\bar{L}}_{T}=\frac{\sum_{j=1}^{n}{\left[\sum_{i=1}^{N}L_{i}l_{i}\right]}_{j}}{\sum_{j=1}^{n}{\left[\sum_{i=1}^{N}l_{i}\right]}_{j}}\;,$$

where l represents the track length of a particle in the given volume. $L_{i}$ is the total electronic stopping power evaluated at each particle step i, calculated using the method *ComputeElectronicDEDX()* of the Geant4 *G4EmCalculator* class. The *G4EmCalculator* calculates restricted and total stopping power and CSDA range via access to precomputed tables or by recomputation of corresponding values. The index i iterates over the total number of steps N taken by a specific particle within the considered volume, while the index j iterates over all primary and secondary particles traveling within that volume.

#### 5.2.2 Results

**FIGURE 23:**
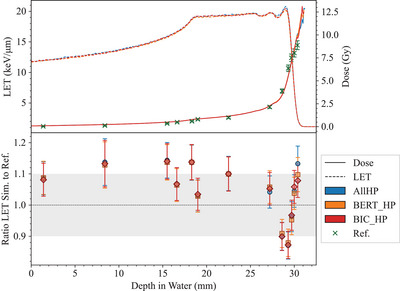
${\bar{L}}_{T}$ curves obtained with Geant4 11.1, when using *QGSP*_*BIC*_*HP* (red), *QGSP_BERT_HP* (orange), and *QGSP_BIC_AllHP* (blue), against $y_{F}$ values obtained experimentally (Petringa et al.^24^). $L_{T}$ uncertainties have been calculated as the standard deviation of 10 simulations executed with different seeds, and they are of the order of 1%. The shadowed area represents an agreement within 10%.

Figure 23 shows the comparison of the track‐averaged LET curve (${\bar{L}}_{T}$), obtained with *QGSP*_*BIC*_*HP*, *QGSP_BERT_HP*, and *QGSP_BIC_AllHP*. The $L_{T}$ uncertainties have been calculated as the standard deviation of 10 simulations executed with different seeds, and they are of the order of 1%. The simulation results are compared to the reference $y_{F}$ values. It is possible to observe that the Geant4 simulations agree within 15% with the reference data. There is no significant difference in the results due to the choice of Geant4 physics lists, among those under study.

### 5.3 In vivo PET test for carbon and oxygen ion therapy

This test has been introduced in the G4‐Med benchmarking system relatively recently and is documented in Chacon et al.^26^ and Chacon et al.^27^ It quantitatively assesses the capability of Geant4 in predicting positron‐emitting fragment production using three hadronic inelastic fragmentation models: BIC, QMD, and INCL.

Experimental data of the absolute yields of positron‐emitting fragments (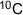, 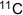, and 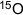), generated during the irradiation of gelatin, PMMA, and polyethylene block phantoms with 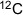 and 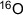 ion beams, serve as reference data. These beams, with energies equal to 148.5, 290.5, and 350 MeV/u for 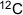 and 148 and 290 MeV/u for 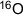, were measured using the OpenPET scanner at HIMAC, Japan, as detailed in Chacon et al.^26^ and Akamatsu et al.^83^

The focus is on the comprehensive comparison of simulated positron yields against experimental measurements across three key regions: entrance, build‐up and Bragg peak, and tail regions.

The experimental set‐up, described in Chacon et al.,^26^ involves irradiating phantoms made of PMMA, polyethylene, or gelatin, using monoenergetic carbon and oxygen ion beams as listed in Table 6. The PMMA chemical composition is $C_{5}H_{8}O_{2}$, with a density of (1.189 ± 0.001) g/${\text{cm}}^{3}$ while the polyethylene composition is $C_{2}H_{4}$ with a density of (0.939 ± 0.001) g/${\text{cm}}^{3}$. The density of gelatin is (1.001 ± 0.002) g/${\text{cm}}^{3}$ and it is distilled water (made with Milli‐Q). No detectable/trace elements other than H and O are assumed to be present.

**TABLE 6:** Beam parameters for each ion species and energy.

Incident ion	Energy (MeV/u)	$\sigma_{x}$ (mm)	$\sigma_{y}$ (mm)	Beam flux (pps)
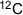	148.5	2.77	2.67	$1.8\times 10^{9}\pm 3.8\times 10^{7}$
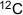	290.5	3.08	4.70	$1.8\times 10^{9}\pm 6.4\times 10^{7}$
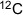	350	2.50	2.98	$1.8\times 10^{9}\pm 4.6\times 10^{7}$
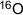	148	2.79	2.89	$1.1\times 10^{9}\pm 2.8\times 10^{7}$
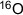	290	2.60	4.90	$1.1\times 10^{9}\pm 7.0\times 10^{7}$

*Note*: All beams had an energy spread of 0.2% of the nominal energy. The uncertainty of the beam energy is estimated to be ± 0.5 MeV/amu while the uncertainty affecting $\sigma_{x}$ (mm) and $\sigma_{y}$ (mm) is 0.05 mm. Ninety‐five percent confidence intervals are listed for beam flux.

Positron annihilation profiles were acquired using a DOI‐PET scanner prototype from QST,^83^ with subsequent decomposition into contributions from 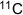, 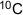, and 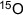 by fitting the time activity curve, voxel by voxel, at the end of the irradiation period. The profile is taken using the full width at tenth maximum (FWTM) of the beam, parallel to the path of the beam in the phantom.

#### 5.3.1 Simulation set‐up

The *in vivo PET* test models the experimental setup as accurately as possible. The selection of physics models followed the configurations specified in the Geant4 advanced hadrontherapy example. The physics lists is *QGSP_BIC_HP* with *Opt3* as EM physics constructor. *Opt3* was chosen in this specific test because it is computationally faster than *Opt4*, without affecting the accuracy of the calculation of the physical quantity under study, the positron yield, which depends on the modeling of the nuclear fragmentation and radioactive decay, when considering that *Opt3* and *Opt4* use the same heavy ion ionization model.

The nuclear fragmentation is modeled with BIC, QMD, and INCL. The heavy ion beams are modeled as two‐dimensional Gaussian pencil beams, with same sigmas (σ) as observed experimentally and detailed in Table 6. An air gap of 1.75 m separates the beam's origin from the phantom's surface to replicate the experimental conditions as accurately as possible. Phantoms, constructed in dimensions of 100 × 100 × 300 ${\text{mm}}^{3}$, are composed of either polyethylene, polymethyl methacrylate (PMMA), or gelatin housed in an open‐top PMMA container. The gelatin phantom features a 4 mm wall thickness and is filled with water. All material properties are defined based on the NIST definitions.

Each simulation configuration is executed with $2\times 10^{7}$ histories. The identification of positron‐emitting fragments is done tracing the origins of annihilating positrons. The spatial and temporal coordinates, alongside the parent ID of each positron annihilation, is recorded with a spatial resolution of 1.5 ${\text{mm}}^{3}$ across the entire phantom, reflecting the capabilities of the OpenPET system for image reconstruction. A Gaussian filter, characterized by a 2.6 mm FWHM, is applied to emulate the point spread function of the PET imaging system. Line profiles, extracted at the FWTM of each beam, traverses the phantom along the beam path. These profiles are normalized to the number of incident particles simulated. Data are grouped into 20 batches for statistical analysis, with the calculation of mean and standard deviation for each. This approach yields a statistical confidence level of 10% in the mean/standard deviation across the majority of the beam's trajectory.

The results are analyzed in three regions, entrance, Bragg peak, and tail. The entrance and the Bragg peak regions are separated by the proximal edge in the z dimension (along the path of the beam), defined as the first point at which the dose deposited along the central axis exceeds the entrance plateau dose by more than 5% of the difference between peak dose and the entrance plateau dose. The Bragg peak and the tail regions are separated by the distal edge in z, defined as the last point at which the deposited dose is greater than 5% of the absolute peak dose value. The simulations are performed with Geant4 11.1.

#### 5.3.2 Results and discussion

**FIGURE 24:**
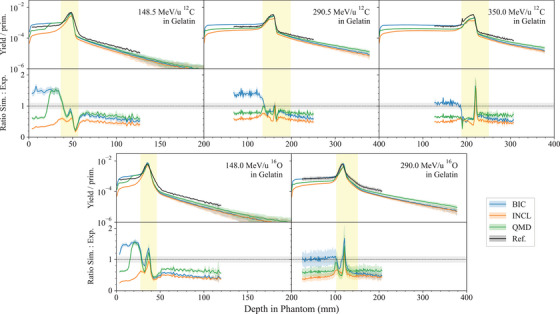
Top row: positron yield per incident 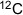 ion, with energy 148.5 MeV/u (left), 290.5 MeV/u (middle), and 350 MeV/u (right). Bottom row: 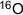 ion, with energy 148 MeV/u (left) and 290 MeV/u (right). The positron yield is plotted with respect to the depth in the gelatin phantom. The yellow areas indicate the Bragg peak regions. The bottom plots in each row show the ratio between simulated and experimental data. The shadowed area represents an agreement within 10%, while the uncertainties (2σ) affecting the simulation results are shown as a shade around the curve. Experimental measurements from Chacon et al.^26^

**FIGURE 25:**
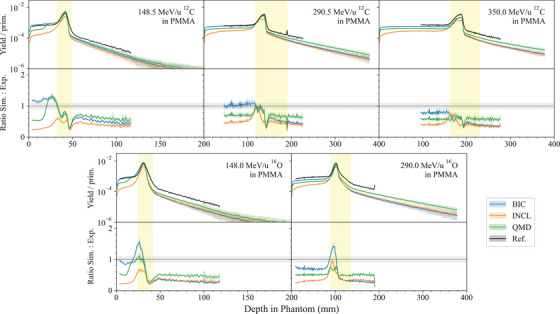
Top row: positron yield per incident 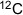 ion, with energy 148.5 MeV/u (left), 290.5 MeV/u (middle), and 350 MeV/u (right). Bottom row: 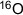 ion, with energy 148 MeV/u (left) and 290 MeV/u (right). The positron yield is plotted with respect to the depth in the PMMA phantom. The yellow areas indicate the Bragg peak regions. The bottom plots in each row show the ratio between simulated and experimental data. The shadowed area represents an agreement within 10%, while the uncertainties (2σ) affecting the simulation results are shown as a shade around the curve. Experimental measurements from Chacon et al.^26^

**FIGURE 26:**
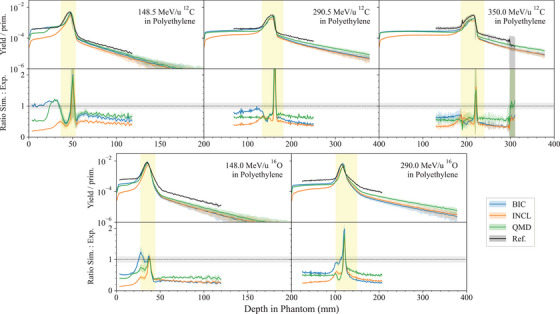
Top row: positron yield per incident 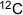 ion, with energy 148.5 MeV/u (left), 290.5 MeV/u (middle), and 350 MeV/u (right). Bottom row: 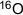 ion, with energy 148 MeV/u (left) and 290 MeV/u (right). The positron yield is plotted with respect to the depth in the polyethylene phantom. The yellow areas indicate the Bragg peak regions. The bottom plots in each row show the ratio between simulated and experimental data. The shadowed area represents an agreement within 10%, while the uncertainties (2σ) affecting the simulation results are shown as a shade around the curve. Experimental measurements from Chacon et al.^26^

Figures 24, 25, 26 show the positron emission yield for the incident 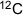 and 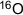 ion beams in the gelatine, PMMA, and polyethylene phantoms, respectively, calculated with Geant4 11.1. The plots have inserts showing the ratio of the Geant4 simulated and experimental data.

There can be observable differences between Geant4 simulations and reference data depending on the irradiation configuration and specific regions of the signal (e.g., entrance, Bragg peak, or distal regions). Table 7 summarizes the results for the tests performed with Geant4 11.1 in terms of MRE and NMAE to identify the fragmentation model that aligns best with the experimental data used in this study. The results are presented specifically for Geant4 11.1 because this test is newly introduced in the G4‐Med benchmarking system and has not been evaluated with earlier Geant4 versions. A more detailed discussion of localized discrepancies and comparisons across different versions of Geant4 can be found in Chacon et al.^27^

**TABLE 7:** Results obtained with Geant4 11.1, summarized using MRE and NMAE metrics (see Section 2).

Target material	Particle	Energy (MeV/u)	Entrance	Build‐up and Bragg peak	Tail region
Gelatin	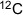	148	QMD	QMD/BIC	QMD
	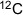	290	QMD	QMD	QMD
	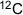	350	BIC	BIC	QMD
	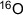	148	QMD	QMD/BIC	QMD
	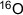	290	BIC	BIC	QMD
PMMA	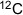	148	BIC	BIC	QMD
	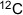	290	BIC	BIC	QMD
	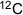	350	BIC	BIC	QMD
	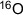	148	BIC	QMD	QMD
	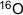	290	BIC	QMD/BIC	QMD
Polyethylene	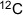	148	BIC	QMD/BIC	QMD
	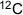	290	BIC	BIC	QMD
	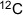	350	QMD/BIC	QMD/BIC	QMD
	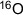	148	BIC	BIC	QMD
	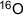	290	BIC	BIC	QMD

The results, including the MRE and NMAE analysis, show that the INCL has the least agreement against the experimental measurements. The BIC provides an overall better agreement for the entrance and Bragg peak region, while the QMD outperforms the BIC in the distal region (tail).

### 5.4 Regression testing results

#### 5.4.1 67.5 MeV proton Bragg peak in water

This test, documented in detail in Arce et al.^13^ and in Faddegon et al.,^28^ calculates the Bragg peak in water of protons of mean energy 67.5 MeV and a gaussian energy spread of 0.4 MeV. The beam is incident on a water phantom, after traversing a tantalum (Ta) foil of either 101.6 or 381 μm thickness.

The physics list includes one of the EM constructors, *Livermore*, *Penelope*, *Opt3*, or *Opt4*, coupled with hadronic physics, which is modeled by means of *QGSP_BIC_HP*.^13^, ^84^

The simulation results are compared to experimental measurements documented in Faddegon et al.,^28^ in terms of range $R_{80}$ (corresponding to the 80% Bragg peak distal fall‐off), and spread σ (indicated here as $\sigma^{*}$ for clarity) of a Gaussian describing the width of the Bragg peak as defined in Bortfeld.^85^ The $R_{80}$ and spread $\sigma^{*}$ are obtained from the fit of an analytical function reported in Bortfeld.^85^ For the measured data, uncertainties include both experimental uncertainties and fitting errors. For the Monte Carlo simulation, uncertainties include statistical (evaluated by executing multiple times the simulation with different random seeds) and fitting errors and are reported at 1 standard deviation.

**FIGURE 27:**
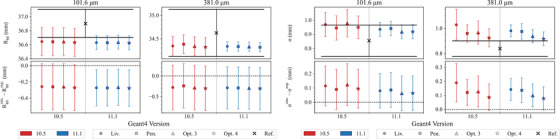
Comparison of $R_{80}$ and Gaussian spread $\sigma^{*}$ between Geant4 10.5/11.1 predictions and reference data, for a 67.5 MeV proton beam incident on a water phantom. Reference data from Faddegon et al.^28^

Figure 27 shows the $R_{80}$ and the spread σ. It can be observed that, while there is no difference between the two releases of Geant4 in terms of $R_{80}$ (agreement within 2σ with the reference data), there is a slight improvement in Geant4 11.1 in terms of gaussian spread $\sigma^{*}$. In the case of the 101.6 μm thick Ta target, the difference of Gaussian spread between simulations and reference data is below 0.10 and 0.14 mm for Geant4 11.1 and 10.5, respectively. In the case of the 381.0 μm thick Ta target, the difference of Gaussian spread between simulations and experiments is below 0.15 and 0.2 mm for Geant4 11.1 and 10.5, respectively. *Opt3* and *Opt4* EM constructors provide the best agreement against reference data in the case of Geant4 11.1. The improvement observed in Geant4 11.1 maybe be due to the ICRU90 adoption to calculate the low energy proton stopping powers in water.

#### 5.4.2 Light ion Bragg peak curves

This test, documented in detail in Arce et al.,^13^ is aimed to compare $R_{82}$ (range of heavy ions at 82% dose fall‐off) in water, between Geant4 and experimental measurements performed at GSI,^86^ for proton and 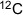 beams with energies of interest for hadron therapy (beam ranges are below 30 cm in water). The EM physics constructors under investigation are *Opt3*, *Opt4*, *Livermore*, and *Penelope*, while the hadronic physics component is defined, for all the simulations, with *QGSP_BIC_HP*, similarly to what done in Arce et al.^13^

**FIGURE 28:**
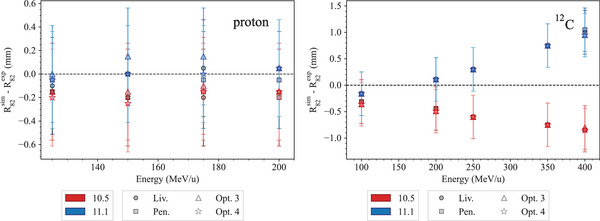
Difference of $R_{82}$ between Geant4 simulations and reference data.^86^ The differences are plotted against the energy of the incident particles (protons and 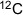). The error bars are calculated at a 95% confidence level.

Figure 28 reports the differences of $R_{82}$ between Geant4 calculations and experimental measurements, for incident protons and 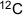 ions. In the Bragg curves, the statistical uncertainty of the dose was calculated following the history‐by‐history method described by Walters et al.^52^ The number of histories was such that the uncertainty of the dose scored at maximum and distal part (>50% dose) was below 1.5%. It was then verified that the uncertainty propagated to the depth $R_{82}$ was approximately below the scoring cell thickness (50 μm) at 1 sigma level. Thus, the uncertainty (1σ) affecting $R_{82}$ was defined to be ±50 μm. This happens thanks to the sharp dose gradients observed at the distal fall‐off. For example, for 200‐MeV protons, the dose fall‐off from 85 to 80% happened in about 300 μm, being the worst‐case scenario for protons. As for carbon ions, at 400‐MeV dose fall‐off from 90 to 70% was observed in about 400 μm. As a result, the experimental uncertainty is 200 μm and the statistical uncertainty of the simulation results is 50 μm.

Taking into account the uncertainties affecting the results, it is possible to observe a general improvement in the calculation of the position of the Bragg peak of approximately 0.2 mm for incident protons in Geant4 11.1.

In the case of incident carbon ions, while Geant4 10.5 tends to underestimate the position of $R_{82}$, Geant4 11.1 overestimates it. Geant4 11.1 predicts more accurately the position of the Bragg peak up to 250‐MeV/u incident energy ($R_{82}^{sim}-R_{82}^{exp}$ is within 0.3 and −0.6 mm for Geant4 11.1 and 10.5, respectively). For higher energies, the absolute difference between simulated and experimental $R_{82}$ is very similar for the two releases of Geant4. In the case of 400 MeV/u, the highest incident energy under study, the difference between simulated and experimental $R_{82}$ is approximately 0.85 and 1 mm for Geant4 10.5 and 11.1, respectively. There is no difference in the results due to the adoption of *Livermore*, *Opt3*, *Opt4*, and *Penelope* EM constructors, because the model of heavy ion ionization process is the same in all these EM constructors.

#### 5.4.3 Neutron yield with protons and ions in thick targets

This test, described in detail in Arce et al.,^13^ calculates the neutron yield in thick targets, produced by incident protons with energy 113 and 256 MeV and 290 MeV/u 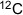 ions. The simulation results are compared to experimental measurements documented in Meier et al.^87^, ^88^ for protons and from Satoh et al.^89^ for carbon ions interacting in water. Table 8 reports the radius, thickness, and material of each target bombarded with either protons or 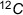 ions. The physics lists under study are *QGSP_BIC_HP* and *Shielding*.

**TABLE 8:** Target dimensions, material, and density for each proton energy and carbon ion beam configuration.

Material	Radius (cm)		Thickness (cm)		Density (${\text{g/cm}}^{3}$)
		Protons			
	113 MeV	256 MeV	113 MeV	256 MeV	
Aluminum	3.65	8.0	4.00	20.0	2.699
Carbon	3.65	8.0	5.83	30.0	1.867
Iron	3.65	8.0	1.57	8.0	7.867
		Carbon ions	(290 MeV/u)		
G4_WATER	—	—		18.0	1.0

*Note*: Table from Arce et al.^13^

The neutron yield per incident primary particle per steradian per energy (equally spaced logarithmic energy intervals) is scored at specific angular bins; for proton beams, 7.5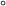, 30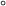, 60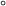, and 150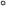 for 113 MeV and 30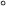, 60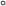, 120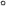, and 150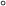 for 256 MeV; 15
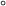
–90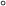 at 15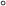 angular steps for carbon ions. Its statistical uncertainty has been calculated by repeating the same simulation with different random seeds and calculating the mean and standard error. The statistical uncertainty of the simulation results is a lot smaller than the experimental uncertainty, which ranges from 7 to 20% and it is dominated by the detector efficiency and cross section data used for the derivation of neutron yields Meier et al.^90^

**FIGURE 29:**
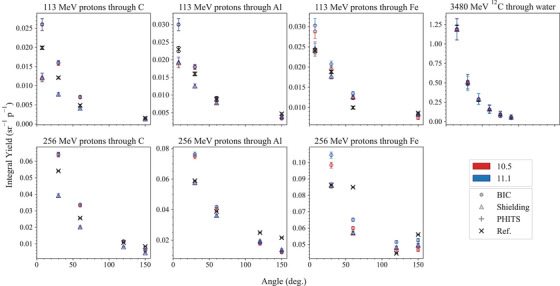
Integral neutron yields with respect to the angle of emission of neutrons, calculated with Geant4 and compared against reference data from Meier et al.^87^, ^88^ for protons and from Satoh et al.^89^ for incident carbon ions. The error bars are within the symbols.

Figure 29 shows the integral neutron yields with respect to the angle of emission of neutrons, calculated in the set of configurations listed in Table 8. Difference between Geant4 10.5 and 11.1 releases is observed only for protons incident on a Fe target, when using *QGSP_BIC_HP*. No difference is reported when using the *Shielding* physics list.

**FIGURE 30:**
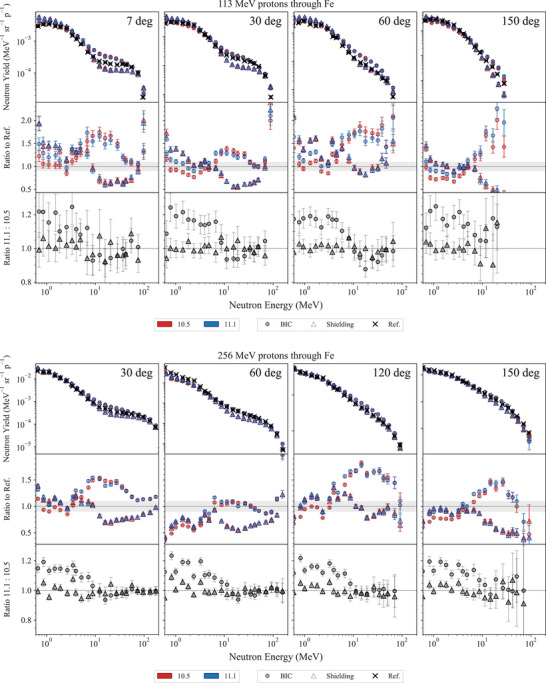
Neutron yields produced by protons in a Fe thick target. Top plot: 113 MeV protons incident on a 1.57 cm thick Fe target. Bottom plot: 256 MeV protons incident on a 8.0 cm thick Fe target. In each plot, top row: neutron yield; second row: ratio of Geant4 results and reference data; third row: ratio of the results obtained with Geant4 11.1 and 10.5. The shadowed area represents an agreement within 10%. When not visible, the error bars are within the symbols. Reference data: Meier et al.^87^, ^88^

When considering the differential neutron yield cross sections, there are differences between the two releases of Geant4 only in the case of protons incident on a Fe target, shown in Figure 30. Differences up to 20% between the two releases of Geant4 are observed in the calculation of the neutron yield below 10 MeV neutron energy, when using *QGSP_BIC_HP*.

This is the only test in the G4‐Med suite that benchmarks Geant4 in terms of neutron production and the results of the regression testing show that more tests are needed to monitor neutron production (in addition to neutron physics).

#### 5.4.4 Fragmentation of a 400 MeV/u ion beam in water

This test, published in Bolst et al.^29^ and described in detail in Arce et al.,^13^ benchmarks Geant4's modeling of the fragmentation process against experimental measurements of a 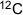 ion beam incident upon a water target performed at GSI.^91^

A 400 MeV/u 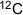 ion pencil beam, with an energy Gaussian spread of 0.15% and a FWHM of 5 mm, is incident on a water phantom with different thicknesses (59, 159, 258, 279, 288, 312, and 347 mm). For each water thickness, secondary fragments and their energy spectra are recorded at different angles at a distance of about 3 m after the water thickness. The physical quantities compared by the benchmark are the yield, angular, and kinetic energy distributions of light fragments, from hydrogen to boron. With the six water thicknesses and five fragment elements, a total of 35 angular distributions and 151 energy distributions are compared between experiment and simulation.

The *QGSP_BIC_HP* physics list is used to model particle interactions. The Geant4 EM constructor *Opt4* is adopted to model the electromagnetic interactions. The tests are performed changing the model of hadronic inelastic scattering of heavy ions: BIC, QMD, and INCL.

**FIGURE 31:**
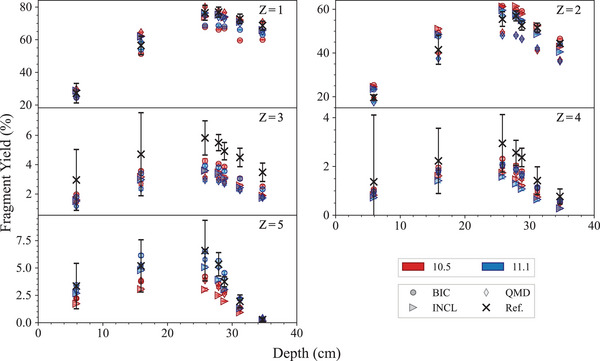
Fragment yield calculated with respect to depth. Experimental data from Haettner et al.^91^ The error bars of the simulation results are within the symbols.

**FIGURE 32:**
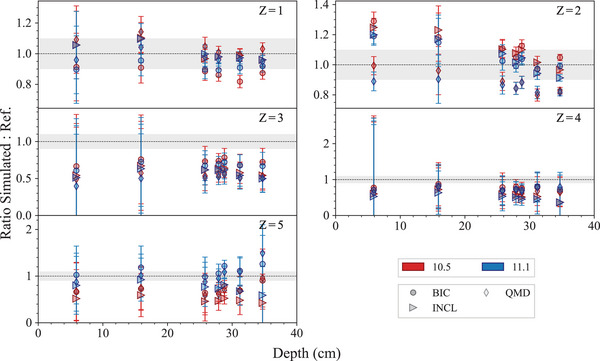
Ratio of Geant4 simulation results and reference data in terms of fragment yields. Experimental data from Haettner et al.^91^ The shadowed area represents an agreement within 10%.

**FIGURE 33:**
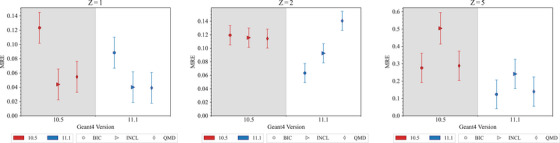
Mean relative error (MRE) calculated for H (left), He (middle), and B (right), for the calculation of the fragments yields. The MREs of fragment yields are calculated by averaging the ratio of experiment and simulation for all six water thicknesses for the particular fragment species. Here, the reported uncertainties correspond to 1σ are provided as 1σ (see Section 2.1).

Figure 31 shows the fragment yield calculated with respect to the depth in water when using Geant4 10.5 and 11.1, while Figure 32 plots the ratio of simulation results and reference data and Figure 33 reports the MRE. The simulation error bars represent the statistical uncertainty when generating a total of 10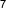 primary 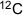 ions for each water thickness. The error bar sizes were calculated by dividing the results into 10 groups and calculating the associated standard deviation.

When considering BIC, Geant4 11.1 provides results that, overall, agree better with the reference data for H, He, and B fragment yields, when compared to Geant4 10.5 (see Figure 33). In particular, the MD with the reference data goes from about 20 to 10% for H, from about 30 to 20% for He, from about 40 to 20% for B, when moving from Geant4 10.5 to 11.1. In the case of INCL, Geant4 11.1 shows a better overall agreement against reference data in the case of He (MD of about 25%–20% for Geant4 10.5 and 11.1, respectively) and B fragments (MD of about 60 and 40% for Geant4 10.5 and 11.1, respectively). In the case of the QMD model, Geant4 10.5 agrees better with the experimental results in the case of the He fragment at shallower depths (MD of about 5 and 10% within 16‐cm depth in water, for Geant4 10.5 and 11.1, respectively). An overall better agreement with Geant4 11.1 has been obtained for QMD for the B fragment yield (MD of about 40 and 15% for Geant4 10.5 and 11.1, respectively, down to a depth of 30 cm), similarly to the other Geant4 fragmentation models. There is no change between Geant4 10.5 and 11.1 for the Li and Be yields, for any of the Geant4 fragmentation models.

**FIGURE 34:**
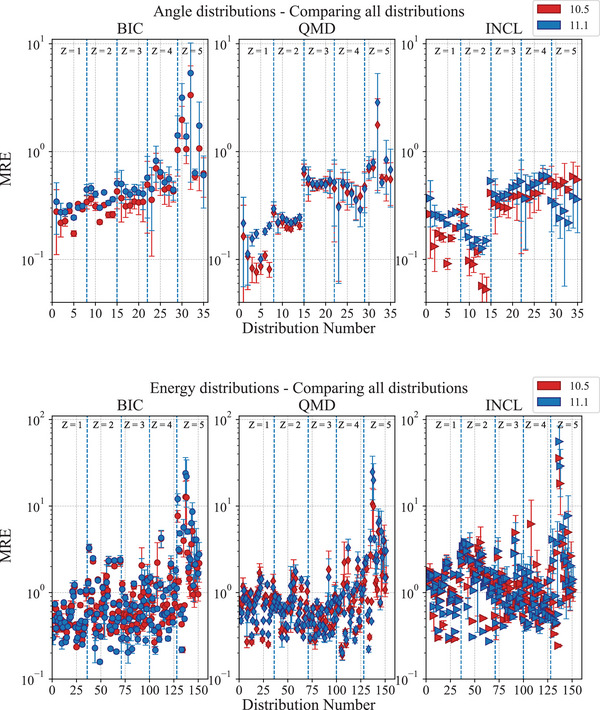
Mean relative error (MRE) calculated for the angular (top) and kinetic energy (bottom) distributions of fragments, for both Geant4 10.5 (red) and 11.1 (blue). The MRE is calculated for individual distributions (35 angular and 151 energy distributions). Here, provided as 1σ (see Section 2.1).

Figure 34 shows the MRE of the angular and kinetic energy distributions, calculated for the light fragments, at different depths in the water medium, for both Geant4 10.5 and 11.1. It is possible to observe that, for both BIC and INCL, there is a slightly less overall agreement in terms of angular distribution of the fragments with Geant4 11.1. Instead, similar agreement against the reference data is obtained for the two releases of Geant4 in terms of kinetic energy distributions.

## 6 GEANT4‐DNA BENCHMARKING TESTS

The Geant4‐DNA package is extensively used in radiobiological studies; therefore, we felt the need to include new tests in the G4‐Med benchmarking suite, to monitor the evolution of Geant4‐DNA physics, physico‐chemical and chemical stage simulation capabilities. So far, we have included three tests to benchmark Geant4‐DNA physics (*Low energy electron Dose Point Kernels* and *microdosimetry* tests) and the physico‐chemical/chemical stages of particle interactions in the *Chemistry* test.

### 6.1 Geant4‐DNA physics constructors

The Geant4‐DNA extension of Geant4 has been providing a range of processes and physical models since 2007. It focuses on simulating particle interactions with liquid water, which is the primary component of the biological environment. This is particularly relevant for studying the impact of ionizing radiation on biological targets at the DNA level.^92^, ^93^, ^94^, ^95^, ^96^, ^97^ These processes and models apply to electrons, protons, neutral hydrogen atoms, helium nuclei, and their charge states, as well as to a number of incident ions.

The processes to which electrons are subjected in the energy range associated to bio‐medical physics applications include ionization, electronic excitation, vibrational excitation, elastic scattering, and dissociative electron attachment. For protons, neutral hydrogen atoms, helium nuclei, and their charge states, ionization, electron excitation, and elastic scattering are available. For ions heavier than helium, only ionization is currently taken into account. These processes are described by alternative models, following different approaches (theoretical, semi‐empirical) and covering different energy domains. They are either analytically coded or can use cross section interpolation.

For ease of use, the processes and models are grouped into alternative Geant4‐DNA physics constructors. The existence of different physics constructors in Geant4‐DNA reflects the continuous efforts to be able to perform track structure simulations for electrons in condensed media with improved accuracy and reduced computational effort. Unfortunately, the absence of experimental data in the liquid‐water phase does not allow them to be validated, but only benchmarked against other in silico calculations. Three alternative constructors are tested in this work: Geant4‐DNA *Option2*, *Option4*, and *Option6*. Each one of these three constructors contains different physics process models for electron transport but a unique model for proton, helium, and ion processes. The emphasis given on electrons stems from the fact that they exist as secondaries from the ionization of atoms of the medium under study and are responsible for the spatial energy deposition of radiation in matter.

Both Geant4‐DNA *Option2* and *Option4* physics constructors (called here *Geant4‐DNA‐Opt2* and *Geant4‐DNA‐Opt4*) are based on the dielectric theory of inelastic scattering, where condensed phase effects are included through the use of a material‐dependent function for the computation of the corresponding cross sections (ionization, electronic excitation) for electrons. *Geant4‐DNA‐Opt4* is an improved version of *Geant4‐DNA‐Opt2* which, among other improvements, uses a refined partitioning algorithm, leading to higher ion‐pair energies, smaller penetration distances, and less diffused dose‐point kernels, and performs simulations in a more accurate manner at the lower energy range.^98^ However, in its publicly available version, it has a limited energy range of applicability compared to *Geant4‐DNA‐Opt2*. An extended relativistic version of *Geant4‐DNA‐Opt4* has recently been developed and will be available in a future version of Geant4‐DNA in order to replace *Geant4‐DNA‐Opt2*.^99^ The Geant4‐DNA *Option6* physics constructor, called here *Geant4‐DNA‐Opt6*, is a re‐engineering of the original CPA100 code for electrons.^100^ It adopts the Binary Encounter Bethe formalism^101^, ^102^ for the analytical computation of ionization cross sections and the dielectric response function formalism for the computation of electronic excitation. The three Geant4‐DNA physics constructors under study cover a different energy range of applicability for electrons (7.4 eV to 1 MeV for *Geant4‐DNA‐Opt2*, 10 eV to 10 keV for *Geant4‐DNA‐Opt4* and 11 eV to 255 keV for *Geant4‐DNA‐Opt6*) and, for convenience, since Geant4 version 11.1, the default high energy limits of *Geant4‐DNA‐Opt4* and *Geant4‐DNA‐Opt6* for electrons have been extended up to 1 MeV using the inelastic cross sections of the *Geant4‐DNA‐Opt2* constructor. Different approaches are provided for the implementation of elastic scattering, which is of critical importance in the spatial deposition of energy because of the deflections suffered by the electrons, in the three constructors. All models, including the relevant processes for protons, neutral hydrogen atoms, helium nuclei and their charge states, and ions, are further described in the 2018 Geant4‐DNA review.^95^ Further, the upper energy limit for proton transport was increased from 100 to 300 MeV by incorporating excitation and ionization models based on the relativistic plane wave born approximation (RPWBA).^103^ Geant4 photon processes (photoelectric effect, Compton scattering, gamma conversion, Rayleigh scattering) and models (Livermore, Klein–Nishina, 5D and Livermore, respectively, documented in Section 3.1) are also available in these constructors.

### 6.2 Geant4‐DNA chemistry constructors

Water molecules that have been ionized and excited through interactions with primary and secondary particles participate in a decomposition process through dissociation channels. These energetically charged water fragments undergo a sequence of breakdown processes, rapidly generating primary water radiolysis products such as $H_{3}O^{+}$, 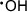, $H^{•}$, ${\mathrm{OH}}^{-}$, and $H_{2}$.^104^ Sub‐excited electrons gradually undergo a thermalization process to reach thermal equilibrium at about 0.025 eV to form solvated electrons.

In Geant4‐DNA, it is assumed that primary water radiolysis species and solvated electrons form within 1 ps, after which they are expected to diffuse through Brownian dynamics and eventually react. The diffusion and reaction of species through these initial heterogeneous distributions (spurs) along the tracks are described by either the step‐by‐step (SBS) approach^104^, ^105^ or the independent reaction time (IRT) approach with two model variants: IRT^106^, ^107^, ^108^ and IRT‐sync.^109^ While SBS transports species in discrete steps (or time steps Δt) through Brownian motion until a chemical encounter defines a reaction, the IRT model tends to directly calculate a reaction probability. The IRT approach assumes that reactions are independent and that the diffusion of reactants from their initial positions to the reaction site is not influenced by other chemical species or volume boundaries. In this context, on the basis of reaction probabilities, the reaction times can be sampled for every potential pair of reactants. These times are then sorted in ascending order, creating a list of potential reactions. Reactions are processed sequentially, starting with those with the shortest reaction time. Products formed from reactions in progress may undergo further reactions with other reactants, thereby being added to the reaction list.

Since IRT models optimize the diffusion actions, this approach has a much better computational performance than the SBS model, especially when dealing with a large number of species. The synchronous IRT (IRT‐sync documented in Tran et al.^109^) model proposes an implementation of the IRT model to allow access to spatial–temporal information at certain times. This implementation uses as the time step the randomly sampled time given by the IRT model until the next expected reaction. In other words, instead of optimizing the time step to the next reaction like the SBS model does, the IRT‐sync model calculates the reaction time directly using the IRT model. This procedure is repeated until the end of the simulation. This means that after each time step, it is necessary to synchronize the time and position of all diffusing species. This is a drawback of the IRT‐sync approach. However, due to synchronization at each time step, IRT‐sync provides users with spatio‐temporal information for all chemical species, which can then be coupled with information on geometric boundaries or the biological target.

The chemistry constructors in Geant4‐DNA specify the dissociation scheme (see Table 9), the chemical reactions and the models involved in water radiolysis. In Geant4 11.1, Geant4‐DNA provides *G4EmDNAChemistry* (*chem_default*), *G4EmDNAChemistry_option1* (*chem_option1*), *G4EmDNAChemistry_option2*(*chem_option2*), and *G4EmDNAChemistry_option3* (*chem_option3*) that deploy different sets of reactions concerning water radiolysis and DNA reactions, accompanied by reaction constant data. While *chem_default*, *chem_option1*, and *chem_option2* constructors use a dissociation scheme following the PARTRAC code,^110^ the chemistry constructor *chem_option3* applies the B1A1 dissociation channel proposed by the TRACs code.^111^, ^112^ *chem_default*, *chem_option1*, and *chem_option2* use the SBS model by default, while in the chemistry constructor *chem_option3*, a user interface allows users to choose either SBS, IRT, or IRT‐sync.

**TABLE 9:** Dissociation schemes and branching ratios available in the four chemistry constructors (*chem_default*, *chem_option1*, *chem_option2*, and *chem_option3*).

			Branching ratios	
Process		Channel	*chem_option3 Shin et al*.^113^	*chem_default* *chem_option1* *chem_option2* Karamitros et al.^104^
Ionization	$H_{2}O^{+}$	$H_{3}O^{+}$ + 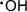	100	100
Excitation	$A^{1}B_{1}$	$H^{•}$ + 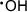	65	65
		$H_{2}O$ + ΔE	35	35
	$B^{1}A_{1}$	$H_{3}O^{+}$ + 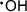 + $e_{aq}^{-}$	50	55
		$H^{•}$ + 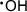	25.35	—
		$H_{2}$ + 2 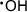	3.25	15
		2$H^{•}$ + O( 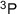 )^a^	3.9	—
		$H_{2}O$ + ΔE	17.5	30
	Rydberg A + B, C + D Diffuse bands,	$H_{3}O^{+}$ + 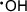 + $e_{aq}^{-}$	50	50
		$H_{2}O$ + ΔE	50	50
Electron capture	DEA	${\mathrm{OH}}^{-}$ + 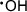 + $H_{2}$	100	100
	Recombination	$H^{•}$ + 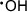	35.75	55
		$H_{2}$ + 2 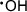	13.65	15
		2$H^{•}$ + O( 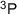 )^a^	15.6	—
		$H_{2}O$ + ΔE	35	30

^a^ Oxygen atom in triplet state P. Geant4 version 11.1 is considered.

### 6.3 Low‐energy electron Dose Point Kernels test

This test is described in detail in Arce et al.^13^ and is of interest for the application of the Geant4‐DNA extension in dosimetry calculations in radiopharmaceutical therapy.^114^, ^115^

Mono‐energetic electrons, with energy 10, 15, and 100 keV, are emitted isotropically into a 4π solid angle from a point source placed in a spherical liquid water (defined as G4_WATER) volume. Radial energy deposition profiles are calculated. $10^{5}$ histories are executed to obtain statistical uncertainties of about 0.3% in the simulation results. The uncertainty has been evaluated calculating average and standard error from a batch of identical simulations with different random seeds.

The test, originally included in the G4‐Med suite to benchmark Geant4 electromagnetic physics constructors using a CH approach,^13^ has been adapted to benchmark Geant4‐DNA physics lists, as track structure codes are recognized to be more accurate to calculate energy deposition distributions in volumes with sub‐cellular dimensions.^116^ Geant4‐DNA physics constructors *Geant4‐DNA‐Opt2*, *Geant4‐DNA‐Opt4*, and *Geant4‐DNA‐Opt6* are tested.

**FIGURE 35:**
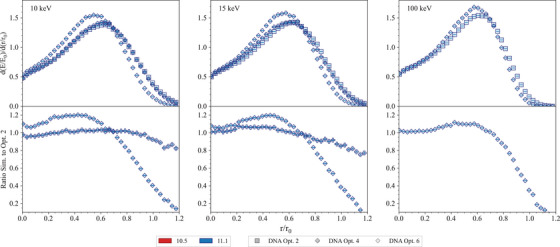
Top plots: Dose Point Kernel in water, obtained for 10, 15, and 100 keV electrons. Bottom plots: ratio of the results obtained with either Geant4‐DNA physics list *Geant4‐DNA‐Opt4* or *Geant4‐DNA‐Opt6* and *Geant4‐DNA‐Opt2*. Results obtained with the *LowEElectDPK* test and Geant4 11.1. The error bars of the simulation results are within the symbols. To note, for 100‐keV electrons, *Geant4‐DNA‐Opt4* provides the same results as *Geant4‐DNA‐Opt2*.

Figure 35 shows the results of the test. We observe that *Geant4‐DNA‐Opt2* and *Geant4‐DNA‐Opt4* provide similar results, while *Geant4‐DNA‐Opt6* estimates the peak of the energy deposition distribution slightly closer to the source of the incident electrons.

### 6.4 The Microdosimetry test

The *Microdosimetry* test is released as the Geant4 extended example *microyz*. The test was already existing in the G4‐Med benchmarking system^13^ but it has been revised and adapted to test *Geant4‐DNA‐Opt2*, *Geant4‐DNA‐Opt4* and *Geant4‐DNA‐Opt6*. It calculates microdosimetric spectra of lineal and specific energy and their corresponding frequency‐weighted and dose‐weighted quantities for the case of monoenergetic electrons traversing liquid water spheres, randomly placed along the track structure of the incident electron in a semi‐infinite water medium.^30^

#### 6.4.1 Simulation set‐up

Monoenergetic electrons are incident on liquid water with energies equal to 100 eV, 1 keV, 10 keV, 100 keV, and 1 MeV, covering both the imaging and therapeutic range, that is, secondaries from hadron therapy or primaries from radionuclide therapy.^117^ The sensitive volume sphere has a diameter of 10 nm, which is a mean distance of interest for studies like double‐strand break induction.^118^ Additionally, spheres with a 100 nm diameter are also investigated here as they are used in biophysical models for radiation action.^119^ Finally, spheres with a 1 μm diameter are considered as they are of interest for tissue‐equivalent proportional counter measurements for radiation protection.^120^

The test is used for regression testing purposes only, to study how changes in the physics models of Geant4 impact the calculation of microdosimetric quantities. In this study, we present results for the dose–mean lineal energy, which is the dose‐weighted probability function of the lineal energy that takes into account the higher dose deposition of the greater valued lineal energies.^121^ In the *microyz* example, the lineal energy is calculated as the ratio of the energy imparted to matter in a given volume (sensitive volume) by a single energy deposition event and the mean chord length of that volume, as defined in Braby et al.^116^ The dose–mean lineal energy is calculated by summing over all the lineal energies $y_{i}$ calculated in each track i, times the contribution of each $y_{i}$ to the dose within the sensitive volume under study.

(6) $$<y_{D}>=\sum_{i=1}^{N}d_{i}y_{i}$$

with $d_{i}=f_{i}y_{i}/<y_{F}>$, $<y_{F}>$ being the frequency‐weighted lineal energy calculated by $<y_{F}>=\sum_{i=1}^{N}f_{i}y_{i}$, $f_{i}$ the probability weighting factor and N the total number of tracks (histories) simulated including primary and secondary particles. The lineal energies $y_{i}$ for each track i are calculated in the Geant4 code by randomly placing a sphere of a user‐defined size along a certain distance from an energy deposit within the track. Details on the algorithm followed in the *microyz* example can be found in Kyriakou et al.^30^, ^117^ The energy cutoff is kept equal to 10 eV for the three physics constructors investigated and the sub‐excitation processes are deactivated.

The statistics for each energy varies according to the incident energy in order to avoid very large simulation times. In consideration of the fact that the microdosimetric simulations using the track structure models of Geant4‐DNA are computationally demanding, they were executed for each energy more than once with different random seeds in order to ensure a statistical uncertainty below 0.1% up to 10 keV, below 0.5% for 100 keV, and below 1% for 1 MeV.

In the *geant‐val* testing suite, the three Geant4‐DNA physics constructors mentioned above are compared up to 10 keV because this is the upper limit of applicability of the specific electron elastic and inelastic models used in *Geant4‐DNA‐Opt4*.^98^

#### 6.4.2 Results

**FIGURE 36:**
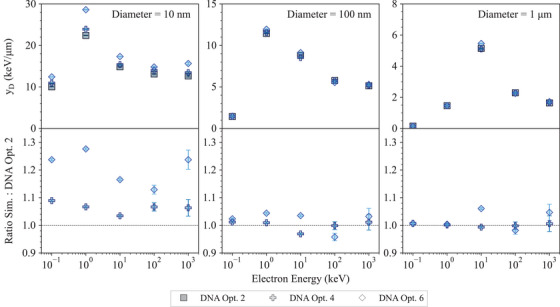
Top plots: Dose–mean lineal energy $y_{D}$ with respect to the kinetic energy of the incident electrons, for a water sphere of 10 nm (left), 100 nm (middle), and 1 μm diameter (right). Bottom plots: ratio of the results obtained with either *Geant4‐DNA‐Opt4* or *Geant4‐DNA‐Opt6* with the default *Geant4‐DNA‐Opt2*. Results obtained with the *microdosimetry* test and Geant4 11.1. When not visible, the error bars are within the symbols.

Figure 36 shows the dose–mean lineal energy, $y_{D}$, as a function of the incident electron kinetic energy. Results are reported for Geant4 11.1. For a water sphere of 10‐nm diameter, differences between the three physics constructors are more pronounced compared to the larger diameters. *Geant4‐DNA‐Opt6* produced $y_{D}$ values higher than those calculated with *Geant4‐DNA‐Opt2*, with a MD of about 28%. These differences are caused by the much different inelastic cross sections of this constructor. Overall, differences between *Geant4‐DNA‐Opt2* and *Geant4‐DNA‐Opt4* are smaller as a result of the similar inelastic models with the differences attributed to the particular ionization and excitation contributions in each model. Additionally, *Geant4‐DNA‐Opt6* contains energy loss for elastic scattering that leads to a decrease of the frequency mean lineal energy values^98^ and especially in the case of smaller diameters (not shown in this study) but does not affect the $y_{D}$. In the case of 100 nm and 1‐μm diameter water sphere, the different Geant4‐DNA physics constructors produce smaller differences; up to 6% for *Geant4‐DNA‐Opt6* and up to 4% for *Geant4‐DNA‐Opt4*, when compared to *Geant4‐DNA‐Opt2*. It must be taken into consideration that above 10 keV for *Geant4‐DNA‐Opt4* and 255 keV for *Geant4‐DNA‐Opt6*, the *Geant4‐DNA‐Opt2* physics constructor is used and we expect that the comparison would be more rigorous if each model was used up to 1 MeV, which will be shown in a future work. In any case, the low (especially the sub‐kilo electron volt) energy behavior affects the energy deposition distribution at sub‐micron volumes.

### 6.5 The chemistry test

The *Chemistry* test is based on the Geant4 extended example *chem6*, which illustrates how to compute radiochemical yields (*G*‐values) versus time or linear energy transfer (LET). The time‐dependent radiolytic yield is often defined as the number of molecules, at a given time, for 100 eV of deposited energy. To reduce simulation execution times, in Geant4‐DNA, the *G*‐values are calculated for a short energy range of the ionizing particle that is referred to as the “track segment G‐value”. Two parameters are used for energy threshold selection, the minimum energy deposition *eLossMin* and the maximum energy deposition *eLossMax*. While the simulation is aborted if the total energy deposition of a simulated event (that is, the primary particle and all associated secondaries) is larger than *eLossMax*, the incident particle is killed if its energy loss exceeds *eLossMin*.^113^ The *G*‐value of each event is calculated and cumulated to calculate N, the total number of events, and $\bar{G}$, the average value of the *G*‐value. Then, the uncertainty affecting the *G*‐value is calculated as $\sqrt{\frac{\frac{\sum_{i}G_{i}^{2}}{N}-{\bar{G}}^{2}}{N-1}}$, where i is an event and $G_{i}$ indicates the *G*‐value calculated in the event i. 10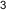 histories are executed in this test.

The *chem6* Geant4 example uses the chemistry constructor *chem_option3*. This constructor offers users an interface to facilitate the addition or removal of chemical reactions or evaluate the influence of chemical reaction–diffusion models, such as the *SBS*, the independent time reaction (*IRT*), and the synchronized independent time reaction (*IRT‐sync*) models, on water radiolysis simulations.

#### 6.5.1 Simulation set‐up

In this test, we perform a simulation to compute time‐dependent *G*‐values for the chemical models *SBS*, *IRT*, and *IRT‐sync*, for 1‐MeV incident electrons with *eLossMin* and *eLossMax* equal to 10 and 10.1 keV, respectively. Table 10 shows the reaction list and reaction constants used in the test.

**TABLE 10:** Reactions and associated reaction rate coefficients (*k*) used by *IRT* and *IRT‐sync* are either partially or totally diffusion‐controlled.

Reaction	*k* $\times 10^{10}$ $M^{-1}s^{-1}$	Partially diffusion‐controlled	Totally diffusion‐controlled
$H^{•}+e_{aq}^{-}+H_{2}O\to {\mathrm{OH}}^{-}+H_{2}$	2.5		X
$H^{•}{+}^{•}\mathrm{OH}\to H_{2}O$	1.55	X	
$H^{•}+H^{•}\to H_{2}$	0.503		X
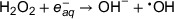	1.1	X	
$H_{3}O^{+}+e_{aq}^{-}\to H^{•}+H_{2}O$	2.11	X	
$H_{3}O^{+}+{\mathrm{OH}}^{-}\to 2H_{2}O$	11.3		X
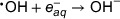	2.95	X	
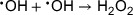	0.55	X	
$e_{aq}^{-}+e_{aq}^{-}+2H_{2}O\to 2{\mathrm{OH}}^{-}+H_{2}$	0.636		X

#### 6.5.2 Results and discussion

**FIGURE 37:**
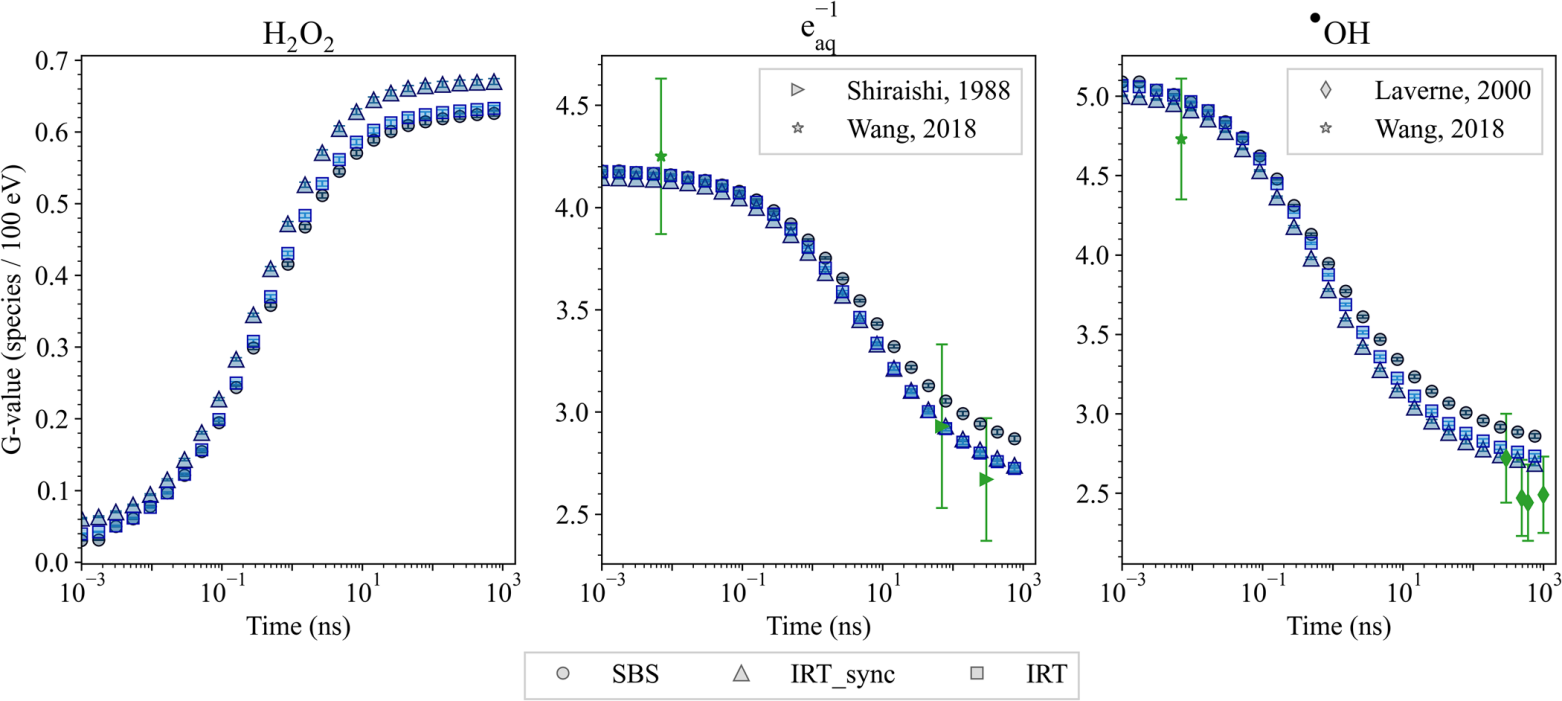
G‐values of $e_{aq}^{-}$, $H_{2}O_{2}$, and 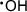 species calculated with the *chemistry* test, using the *SBS*, *IRT‐sync*, and *IRT* models. Results obtained with Geant4 11.1. The error bars of the simulation results are within the symbols.

**FIGURE 38:**
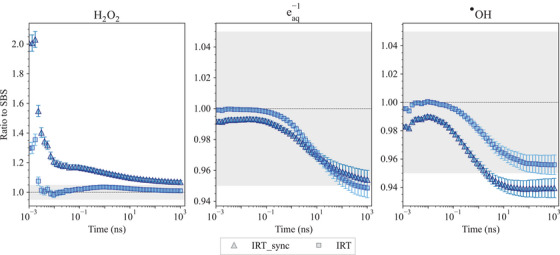
Ratio of G‐values (plotted in Figure 37) obtained with *IRT‐sync* and *IRT models*, when compared to SBS. The shadowed area indicates an agreement within 5%.

Figure 37 shows the *G*‐values of $e_{aq}^{-}$, $H_{2}O_{2}$, and 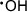 species, calculated using the *SBS*, *IRT‐sync*, and *IRT* models, against available experimental measurements.^122^, ^123^, ^124^ The *G*‐values are calculated with Geant4 11.1. Figure 38 shows the ratio of the *G*‐values calculated with either *IRT* or *IRT‐sync* and *SBS*.

In the case of $H_{2}O_{2}$, both *IRT* and *IRT‐sync* produce higher *G*‐values (about 1.3 and 2 times higher, respectively) at the start of the chemical stage when compared to *SBS*. After that, the difference decreases with time. In the case of $e_{aq}^{-}$, *IRT* produces similar results to *SBS* within approximately 0.1 ns from the start of the chemical stage. After that, it produces lower *G*‐values with a MD of about 5% at 1 μs. *IRT‐sync* underestimates the $e_{aq}^{-}$ *G*‐values of about 1%–5% with respect to the *SBS*. In the case of 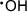 *G*‐values, both *IRT* and *IRT‐sync* produce consistently lower *G*‐values (differences up to approximately 4 and 6%, respectively, when compared to *SBS*).

**FIGURE 39:**
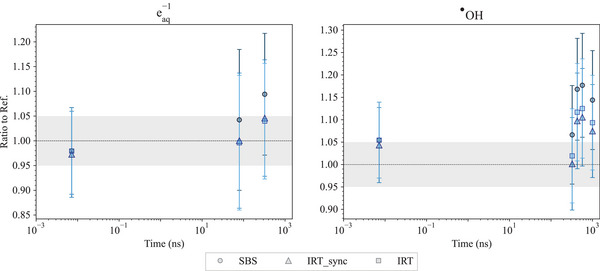
Ratio of G‐values obtained with *SBS*, *IRT*, and *IRT‐sync* (Geant4 11.1) and experimental data.^122^, ^123^, ^124^ The shadowed area indicates an agreement within 5%.

Figure 39 reports the ratio of the results obtained with Geant4 11.1 and the reference experimental data.^122^, ^123^, ^124^ The three chemistry models of Geant4 agree with the reference data within 2σ, apart from the case of 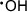 around 400 ns to 1 μs, where Geant4 tends to overestimate the *G*‐value. Nevertheless, provided the few experimental data, it is difficult to draw any conclusion, more reference data are needed.

## 7 COMPUTING PERFORMANCE TEST

The aim of this part of the project is to benchmark the execution times of the main Geant4 physics constructors and lists used within the G4‐Med suite. This is achieved using a simplified Geant4 simulation, described in Section 7.1.

Each simulation configuration of the Geant4 application is executed in sequential mode (i.e., single threaded) on a dedicated computing server, a 2.30 GHz Intel Xeon E5‐2650v3‐based machine maintained by the Centre for Medical and Radiation Physics, University of Wollongong, Australia. Each simulation configuration is repeated 10 times with different random seeds. Then, the average and standard deviation of the execution times are calculated. The computing performance ε is then calculated as $\varepsilon =\frac{T}{T_{ref}}$, where T and $T_{ref}$ are the execution times of the Geant4 simulation for a particular physics lists/constructor and a reference list/constructor, respectively.

### 7.1 Geant4 simulation set‐up

The Geant4 simulation consists of a 30‐cm water cube, a pencil beam is generated at the center of one of the face's surface, just inside the water medium. No particle track information or other physical quantities (for example, energy deposition) are retrieved or stored. The simulation only generates primary particles with a defined energy and direction, and tracks them in the water phantom. The execution time from the start of the first event to the end of the last event is stored. This is to minimize the contribution to the computational time deriving from components other than the physics modeling, for example, geometry operations, retrieval and storage of track and stepping information in Geant4 User Actions. An “event” is the generation of a single primary particle within the simulation geometry and tracking this primary particle as it interacts in the geometry. The end of an event occurs once the primary particle and all secondary particles it creates either exit the simulation's geometry or lose all their kinetic energy. The total number of events chosen by the user is referred to as a “run.”

The quantity compared for these execution times is the *execution time per event*, obtained from dividing the recorded runtime of all the events by the number of events. Configuration runtimes were averaged over 10 separate runs using different seeds, with the standard deviation of the 10 runs used as the uncertainty. The number of primaries generated for each configuration (run 10 times) was chosen such that the runtime would be at least 1000 s and are summarized in Table 11.

**TABLE 11:** Number of primary particles (events) generated for each computation configuration, used to compare the computing performance of Geant4‐DNA physics lists, Geant4 EM physics constructors based on condensed history approach and Geant4 physics lists (with both EM and hadronic physics processes).

Configuration	Number of primary particles
**Geant4‐DNA physics lists**	
1 keV $e^{-}$	$10^{6}$
10 keV $e^{-}$	$10^{5}$
**Geant4 EM physics constructors**	
10 keV $e^{-}$	$10^{8}$
100 keV $e^{-}$	$10^{7}$
1 MeV $e^{-}$	$10^{7}$
10 MeV $e^{-}$	$10^{6}$
**Geant4 EM physics lists (EM + hadronic processes)**	
150‐MeV protons, 0.1 mm cut	$10^{7}$
150‐MeV protons, 1 m cut	$10^{7}$
290‐MeV/u 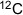 ions, 0.1 mm cut	$10^{5}$
290‐MeV/u 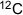 ions, 1 m cut	$10^{6}$

*Note*: Each configuration was run 10 times for statistical calculations, meaning that the total number of primary particles generated is 10 times larger than the ones reported in this table.

#### 7.1.1 Electromagnetic settings

Mono‐energetic electron beams are generated to compare the execution times of both Geant4‐DNA track structure (*Geant4‐DNA‐Opt2*, *Geant4‐DNA‐Opt4*, and *Geant4‐DNA‐Opt6*) and Geant4 CH EM constructors (*Opt3*, *Opt4*, *Livermore*, and *Penelope*).

The energy of the primary electron beams are as follows: 1, 10, 100, 10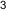, 10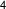 keV. All the Geant4 CH EM physics constructors have a cut of 1 μm and a threshold production of 1 keV, which corresponds to an electron range in water of ∼1 μm. The choice of a 1 keV production threshold is dictated by the fact that this is the minimum recommended energy of the Geant4 EM *Opt3* constructor.

Until version 11.0, simulations with the Geant4‐DNA physics constructors were performed for primary energies of 1 and 10 keV only, due to *Geant4‐DNA‐Opt4* and *Geant4‐DNA‐Opt6* having maximum electron ionization energies of 10 and 255 keV, respectively. Since version 11.1, a combination with Born models (ionization and excitation) extends the maximum energies up to 1 MeV for *Geant4‐DNA‐Opt4* and *Geant4‐DNA‐Opt6*. Conversely, Geant4 CH EM physics constructors are executed only for electron energies of 10 keV and higher due the adoption of the 1‐keV cut.

#### 7.1.2 Hadronic settings

The execution times of the prebuilt Geant4 physics lists for hadronic interactions are compared using a 150 MeV proton and a 290 MeV/u 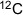 ion beam incident on the 30 cm water cube. In the case of the proton beam, the *QGSP_BIC_HP*, *QGSP_BIC_AllHP* and *QGSP_BERT _HP* physics lists are compared. In the case of an incident 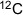 ion beam, the *QGSP_BIC_HP* is adopted. The ion fragmentation is modeled alternatively with BIC, QMD, and INCL. *Opt4* is chosen in all the Geant4 physics lists under study to describe EM physics interactions, as it is deemed to be the most accurate for bio‐medical applications.

Both the proton and 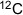 ion simulations are executed with a 0.1 mm and a 1 m cut. The 0.1 mm value represents a more clinically relevant description of the generation of delta electrons in the simulation. The 1 m cut has instead been chosen to minimize the contribution of the EM physics interactions (no generation of delta electrons), to compare the effect of different hadronic physics models in the execution times.

### 7.2 Results and discussion

Execution times using Geant4‐DNA physics constructors, for electron beams with energies 1 and 10 keV in water, are shown in Table 12. For 1 keV electron beams, *Geant4‐DNA‐Opt4* is about 12% slower than *Geant4‐DNA‐Opt2*, while *Geant4‐DNA‐Opt6* is ∼15% faster. At 10 keV, *Geant4‐DNA‐Opt4* becomes significantly more computationally intensive than *Geant4‐DNA‐Opt2*, with ∼70% longer execution time, with *Geant4‐DNA‐Opt6* maintaining a similar ratio with *Geant4‐DNA‐Opt2* at both 10 and 1 keV cases.

**TABLE 12:** Execution time per event calculated with incident electrons, with energy 1 and 10 keV, in a water phantom, for the Geant4‐DNA physics constructors studied in this work.

Energy (keV)	EM Physics constructor	Execution time per event (μs)	$\frac{G4\mathrm{EMco}\mathrm{nstr}\mathrm{uctor}}{\mathrm{Geant}4-\mathrm{DNA}-\mathrm{Opt}2}$
1 keV	*Geant4‐DNA‐Opt2*	(414. ± 3.)·10	1
	*Geant4‐DNA‐Opt4*	(466. ± 3.)·10	1.12 ± 0.01
	*Geant4‐DNA‐Opt6*	(353. ± 2.)·10	0.85 ± 0.01
10 keV	*Geant4‐DNA‐Opt2*	(430. ± 2.)$\cdot 10^{2}$	1
	*Geant4‐DNA‐Opt4*	(744. ± 4.)$\cdot 10^{2}$	1.73 ± 0.01
	*Geant4‐DNA‐Opt6*	(357. ± 4.) $\cdot 10^{2}$	0.83 ± 0.01

*Note*: Results obtained with Geant4 11.1.

The execution times obtained with the Geant4 EM CH approach constructors when simulating electron beams with energy between 10 and 10 MeV are shown in Table 13. For the case of 10 keV electrons in water, *Opt4*, *Livermore*, and *Penelope* have the same computing performance and are about twice slower than *Opt3*. When considering 100 keV electrons, *Opt4* and *Penelope* are about three times slower than *Opt3*, while *Livermore* is about 3.7 times slower. At higher electron beam energies, 1 MeV and above, *Penelope* becomes significantly slower than *Opt4* and *Livermore*. At these higher energies, *Opt4* and *Livermore*'s execution time ratios reduce to ∼2.5× and ∼2.2× at 1 and 10 MeV, respectively, when compared to *Opt3*, while *Penelope* is about four times slower than *Opt3* for both electron energies.

**TABLE 13:** Execution times per event obtained with the simulations modeling 0.10, 0.1, 1, and 10 MeV electron beams in water.

Energy (MeV)	Model	Execution time per event (μs)	$\frac{G4\mathrm{EM}\mathrm{Constructor}}{\mathrm{Opt}3}$
0.01	*Opt3*	23. ± 1.	1
	*Opt4*	45. ± 2.	(2.0 ± 0.2)
	*Penelope*	46. ± 3.	(2.0 ± 0.2)
	*Livermore*	48. ± 1.	(2.1 ± 0.1)
0.1	*Opt3*	48. ± 3.	1
	*Opt4*	143. ± 2.	3.0 ± 0.2
	*Penelope*	145. ± 7.	3.0 ± 0.2
	*Livermore*	178. ± 3.	3.7 ± 0.2
1	*Opt3*	(30. ± 1.)·10	1
	*Opt4*	(75. ± 3.)·10	2.5 ± 0.1
	*Penelope*	(120. ± 10.)·10	3.9 ± 0.4
	*Livermore*	(74 ± 3)·10	2.5 ± 0.1
10	*Opt3*	(27. ± 1.)$\cdot 10^{2}$	1
	*Opt4*	(58. ± 3.)$\cdot 10^{2}$	2.2 ± 0.1
	*Penelope*	(109. ± 4.)$\cdot 10^{2}$	4.1 ± 0.2
	*Livermore*	(59. ± 1.)$\cdot 10^{2}$	2.2 ± 0.1

*Note*: Results shown for the Geant4 condensed history EM physics constructors under study, with Geant4 11.1.

Table 14 show the execution times per event obtained in the case of a 150 MeV proton beam, with the different Geant4 physics lists under study and two different cuts (0.1 mm and 1 m). For both cut sizes, the *BIC_HP* and *BERT_HP* have very similar execution times. *QGSP_BIC_AllHP* is characterized by significantly shorter execution times than *QGSP_BIC_HP* and *QGSP_BERT_HP*.

**TABLE 14:** Execution times per event of the Geant4 physics lists used in this work, for a 150‐MeV proton beam in a water phantom.

Cut	Geant4 physics list	Execution time per event (μs)	$\frac{\mathrm{Geant}4\mathrm{Physics}\mathrm{List}}{\mathrm{QGSP}\_\mathrm{BIC}\_\mathrm{HP}}$
0.1 mm	*QGSP_BIC_HP*	(133. ± 8.)·10	1
	*QGSP_BIC_AllHP*	(31. ± 1.)·10	0.23 ± 0.02
	*QGSP_BERT_HP*	(131. ± 6.)·10	0.99 ± 0.07
1 m	*QGSP_BIC_HP*	425. ± 9.	1
	*QGSP_BIC_AllHP*	47. ± 2.	0.11 ± 0.01
	*QGSP_BERT_HP*	391. ± 9.	0.92 ± 0.03

*Note*: Results obtained with Geant4 11.1.

Comparison of execution times per event obtained with *QGSP_BIC_HP* and changing the ion fragmentation model are shown in Table 15, for a 290 MeV/u 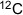 ion beam in water. The simulations with BIC and INCL have similar execution times. In the case of the smaller 0.1 mm cut, the execution time obtained with the QMD fragmentation model is about 40% longer than in the case of BIC and INCL. In the case of the higher cut of 1 m, the execution time obtained with QMD is about four times longer than BIC and INCL. Both BIC and QMD model the interaction of projectile and target nucleons as Gaussian wave functions. However, the reason for the significantly longer QMD execution times is that, compared to BIC, QMD produces wave functions for all target and projectile nucleons in the interaction, while BIC only produce wave functions for *participating* nucleons. Additionally, QMD is time‐dependent while BIC is time‐independent. The INCL model instead describes the wave functions of the interacting nucleons as free Fermi gas in a static potential well, which shows to be computationally similar to the BIC.

**TABLE 15:** Execution times of the Geant4 ion fragmentation models, BIC, INCL, and QMD, for a 290 MeV/u ion beam in water.

Cut	Geant4 ion fragmentation model	Execution time per event (μs)	$\frac{Geant4PhysicsList}{BIC}$
0.1 mm	BIC	(332. ± 9.)$\cdot 10^{2}$	1
	QMD	(469. ± 8.)$\cdot 10^{2}$	1.41 ± 0.05
	INCL	(321. ± 8.) $\cdot 10^{2}$	0.97 ± 0.04
1 m	BIC	(47.0 ± 0.8) $\cdot 10^{2}$	1
	QMD	(188. ± 1.)$\cdot 10^{2}$	4.01 ± 0.07
	INCL	(44. ± 2.)$\cdot 10^{2}$	0.94 ± 0.04

*Note*: Results obtained with Geant4 11.1.

## 8 DISCUSSION OF THE RESULTS

### 8.1 Electromagnetic physics tests

Three new EM physics tests have been introduced in the G4‐Med benchmarking system since our first work documented in Arce et al.^13^ to extend the validation of Geant4 to low‐dose‐rate brachytherapy, MV x‐ray external radiotherapy and electron FLASH radiotherapy, extending the coverage of the G4‐Med benchmarking system to more medical physics application scenarios.

The electromagnetic physics tests of the G4‐Med benchmarking system showed some significant differences between Geant4 10.5 and 11.1 in *Opt3* due to differences in the multiple scattering, documented in Section 3.1. While *Opt3* in Geant4 11.1 provides a better description of electron backscattering (see Section 3.5.1), it is less accurate to describe the transmission of electrons in the forward direction, when compared to *Livermore*, *Penelope*, and *Opt4* (see Section 3.5.2), which then translates in a less accurate calculation of dose (see Section 3.4). Because of this finding, a patch of Geant4 has been released (11.2.1) where the multiple scattering parameters of *Opt3* have been reverted to those of Geant4 10.5. Thanks to this change, the electron transmission and dose calculations with *Opt3* show to improve, but our results indicate that the agreement achieved with Geant4 10.5 cannot still be fully reproduced. Work is under way to understand the source of the observed behavior.

The *MV x‐ray radiotherapy* test (Section 3.3) shows some significant differences in the implementation of the bremsstrahlung model between *Penelope* and *Opt4*, which will be the subject of a detailed investigation.

Based on our findings in the *electron backscattering*, *MV x‐ray radiotherapy*, *Electron FLASH radiotherapy*, *13 MeV electron forward scatter* and *Bremsstrahlung from thick targets* tests, it is recommended to use the EM constructor *Opt4*, which shows to be—overall—the most adequate choice among the available EM constructors for different medical physics application scenarios.

### 8.2 Hadronic physics tests

Three hadronic inelastic scattering tests are currently included in the G4‐Med benchmarking suite.

The regression testing showed that the nucleus–nucleus hadronic inelastic scattering cross sections of incident protons on 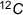, 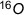, 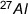, and 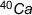 targets changes between Geant4 10.5 and 11.1 for energies below 10 MeV (see Section 4.1.1). This change is due to the change of the Coulomb barrier parameterization in the cross section computation. Geant4 11.1 hadronic cross sections are deemed to be more accurate as they are obtained from tabulation of ParticleHP evaluated data based on the best known experimental data and at higher energies on the Glauber–Gribov theory.^3^

The 300 MeV/u 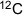 ion charge‐changing cross section test (Section 4.1.3) shows significant differences between Geant4 10.5 and 11.1 in the calculation of the fragmentation yields. Geant4 11.1 describes significantly better the B isotopes yield, with approximately a 20% agreement with the reference data, when compared to Geant4 10.5. Nevertheless, this improvement is accompanied by a worsening of the results for Be production of about 10%–15%. The change in the fragmentation yields may be due to the change of de‐excitation channels of the Fermi Break‐Up model, nevertheless more tailored tests should be performed to confirm this hypothesis. Among the three fragmentation models under study (BIC, QMD and INCL), BIC produces overall the best agreement with the reference data. The results of the 300 MeV/u 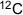 ion charge‐changing cross section test (Section 4.1.3) are coherent with the results of the 62 MeV/u 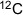 fragmentation test (Section 4.1.2).

In summary, the results show that it is paramount to execute the G4‐Med nuclear fragmentation tests whenever there is a new development in the hadronic physics of Geant4 to monitor closely the evolution of its physics models. In addition, more tests need to be included in the G4‐Med suite to benchmark individual models (e.g. the Fermi Break‐Up model), to cover a wider energy range of carbon ions of interest for hadron therapy, to include more cross sections of interest, for example for accelerator‐based Boron Neutron Capture Therapy, and neutron radiation fields.

### 8.3 Electromagnetic and hadronic physics tests

Two new tests have been included in the G4‐Med benchmarking suite covering two new application scenarios of interest for hadrontherapy, experimental, microdosimetry (Section 5.2) and in vivo PET (Section 5.3).

When considering the G4‐Med tests of interest for proton therapy, the following observations can be made. *QGSP_BIC_HP*, *QGSP_BIC_AllHP*, and *QGSP_BERT_HP* produce the same results when calculating the track‐averaged LET of a 62 MeV SOBP proton beam in water. The agreement with reference data is within 15% (*Hadrontherapy* test, Section 5.2).

The regression testing of *67.5 MeV proton Bragg curves in water* (Section 5.4.1) shows a slight improvement in Geant4 11.1 in terms of spread of a Gaussian describing the width of the Bragg peak, while the results of the *Light Ion Bragg Peak curves* test show that the $R_{82}$ of protons with energy up to 200 MeV in water improves of approximately 0.2 mm in Geant4 11.1. These improvements are ascribed to the ICRU90 adoption to calculate the low energy proton stopping powers in water in Geant4 11.1.

The *Neutron yield of protons with energy 113 and 256 MeV* test (Section 5.4.3) reports that *QGSP_BIC_HP* in Geant4 11.1 produces a higher yield of neutrons (up to approximately 20%) with energy below 10 MeV for protons with 113 and 256 MeV energies, incident on a Fe target. No difference between Geant4 10.5 and 11.1 is found for the lighter targets considered (C and Al) in the irradiation scenarios under study.

When considering the G4‐Med tests of interest for heavy ion therapy, the in vivo PET test (Section 5.3) shows that BIC and QMD are more adequate than INCL to describe the positron yield for incident 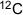 and 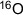 beams in the targets under study (gelatin, PMMA, and polyethylene). Overall, BIC provides better description of the positron yields in the Bragg peak region, while QMD outperforms BIC after the distal edge of the Bragg peak.

The regression testing of the *Light Ion Bragg Peak curves* test show that the $R_{82}$ in water changes between Geant4 10.5 and 11.1 due to the introduction of the Lindhard–Sorensen ion model, available in Geant4 since version 11.0. When compared to Geant4 10.5, Geant4 11.1 produces $R_{82}$ closer to the reference data for 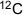 ions with energies up to 250 MeV/u (within 0.3 and 0.6 mm for Geant4 11.1 and 10.5, respectively). Instead, in the case of 400 MeV/u 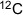 ions, which is the largest energy of the carbon ion beam under study, the difference in the calculation of $R_{82}$ between Geant4 simulation results and experimental data is about 0.85 and 1.0 mm, for Geant4 10.5 and 11.1, respectively.

The *Neutron yield of carbon ions with energy 290 MeV/u* test did not report any change in the neutron production yield of 290 MeV/u 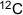 ions in water between Geant4 10.5 and 11.1.

The *Fragmentation of a 400 MeV/u* 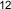 *C ion beam in water* test shows a higher production of the boron fragment, leading to a better agreement with the reference data. This result is consistent with the results of the 300‐MeV/u 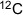 ion charge‐changing cross section test (Section 4.1.3).

Arce et al.^13^ recommended for hadrontherapy simulations the use of *QGSP_BIC_HP* with *Opt4* (*Opt4* is the default EM constructor in *QGSP_BIC_HP* since Geant4 11.0) to describe the EM physics component, as this physics list provides an overall adequate description of the physics involved in hadron therapy, including proton and carbon ion therapy. The same recommendation is given here based on the results of the G4‐Med tests documented in this special report.

To note, for nuclear fragmentation, a novel QMD model^125^ has been introduced in Geant4 11.2, called LiQMD, whose model parameters have been improved for carbon ion therapy. This model will be included in the next future in the G4‐Med benchmarking activity as first results showed a better agreement with experimental reference data for nuclear fragmentation.^125^

It should be noted that the provided recommendations in terms of Geant4 physics list do have some limitations. Only few physical quantities (position and spread of the Bragg peak, neutron yields and fragment yields) in specific irradiation scenarios have been considered. Despite our effort to increase the number of hadronic physics tests (two new tests have been included since Arce et al.^13^), more tests should be added to extend the benchmark further for nuclear fragmentation and to test neutron production and interactions.

The hadronic physics of Geant4 is an active domain of development and the results of the G4‐Med benchmarking system highlight the need to perform regression testing whenever there is a change in the hadronic physics of Geant4 to monitor the evolution of this Monte Carlo simulation code.

### 8.4 Geant4‐DNA tests

Three tests have been introduced in the G4‐Med suite to perform Geant4‐DNA regression testing: the *Low energy electron Dose Point Kernels*, *microdosimetry*, and *chemistry* tests. In summary, the *Low energy electron Dose Point Kernels* and *microdosimetry* tests show that *Geant4‐DNA‐Opt2* and *Geant4‐DNA‐Opt4* produce slightly different results in the physical quantities under study, while more significant differences can be found when these two physics constructors are compared to *Geant4‐DNA‐Opt6*. Differences between Geant4‐DNA physics constructors in dose–mean lineal energy tend to increase for sensitive volume sizes below 100 nm and move toward the smallest differences for the 1 micron volume diameter due to the absorption of most of the sub‐kilo‐electron‐volt electrons within the scoring site. In general, the different cross sections contained in the three different physics constructors are more likely to affect studies concerning subkilo‐electron‐volt energies and smaller scoring volumes as it has already been shown in previous relevant studies, that is, Refs. 117, 119, 126.

The *chemistry test* shows that the *SBS*, *IRT*, and *IRT‐sync* can produce different *G*‐values, depending on the chemical species under study and considered time interval after the start of the chemical stage.

More Geant4‐DNA tests will be added to future versions of the G4‐Med suite for regression testing of physics and chemistry models and for easier comparison to other simulation data or measurements.

### 8.5 Execution times test

Differences in physics models (e.g., analytical or based on data), parameters and data extrapolation techniques have an impact on simulation execution times. Therefore, a test has been added to the G4‐Med benchmarking suite to compare different Geant4 EM physics constructors and physics lists in terms of execution times, in a very simple simulation configuration.

In summary, the results show that, in general, *Geant4‐DNA‐Opt6* is the fastest when compared to *Geant4‐DNA‐Opt2* and *Geant4‐DNA‐Opt4*. When considering Geant4 EM CH approach models under study, in the case of electron beams incident in water with energy from 10 keV to 10 MeV, *Opt3* showed globally to be the fastest Geant4 EM constructor under study, followed by *Opt4* and *Livermore* and, finally, by *Penelope*.

The benchmarking of the execution times of the test with a 150 MeV proton beam in water, of interest for proton therapy, shows that *QGSP_BIC_AllHP* is the fastest Geant4 physics list for proton therapy and that *QGSP_BIC_HP* and *QGSP_BERT_HP* have similar execution times in the specific test under study. In the case of an incident 290 MeV/u 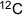 ion beam, BIC and INCL ion fragmentation models show similar execution times, while the QMD is significantly slower.

## 9 CONCLUSIONS

The G4‐Med benchmarking suite was born in 2014 from the effort of an international collaboration, the Geant4 Medical Simulation Benchmarking Group, to validate Geant4 and monitor its evolution for bio‐medical applications. The G4‐Med system was first documented in Arce et al.,^13^ where the results of the tests were reported for Geant4 10.5. In this special report, we describe the evolution of the project in terms of novel tests included in the G4‐Med testing system and the results of the regression testing between Geant4 10.5 and 11.1.

In summary, new tests have been included to benchmark Geant4‐DNA physics models and the capability of Geant4 for external x‐ray and electron FLASH radiotherapy. New hadron therapy tests include Geant4 applications in experimental microdosimetry and in vivo PET.

Overall, the regression testing results showed the importance of the G4‐Med benchmarking system to maintain and improve Geant4 for bio‐medical physics applications. For example, thanks to this project, it was possible to identify problems in the implementation of the multiple scattering of the Geant4 EM constructor *Opt3* in Geant4 11.1 (which was then revised in Geant4 11.2.1). It was then possible to check the impact of novel proton and ion ionizations models in results of interest for hadron therapy and to monitor the evolution of hadronic inelastic cross sections.

The MRE, the NMAE and the MD, which could be applied transparently to any tested physical quantity of the G4‐Med testing suite, proved to be adequate metrics for the regression testing and they provided a consistent analysis of the results.

The regression testing study showed that there can be significant differences between different Geant4 versions, therefore it is recommended to perform in‐house regression testing for the specific application scenario of interest, when moving from an older to a more recent version of Geant4.

For the first time, we reported a comparison of the Geant4 EM physics constructors and physics lists under study in terms of computational times. A simple Geant4 simulation was developed for this purpose, which will be executed for future Geant4 releases.

The following areas of future development of the G4‐Med benchmarking system arose from this work. While the execution of the tests in geant‐val is automatized, the code maintenance, the inclusion of the results in the geant‐val web interface, and the analysis of the results are manual. In order to speed‐up the G4‐Med system and be able to use it more efficiently to monitor the evolution of the Geant4 physics lists frequently (e.g., three/four times per year), the entire G4‐Med system needs to be automatized, from the execution of the individual tests to the analysis of the regression testing results in terms of MRE, NMAE, and MD. This development would allow to monitor more efficiently the evolution of Geant4 and to perform swiftly regression testing studies few times per year before the public release of Geant4, while now, provided our current resources and the status of the developed software tools used in the project, we are able to perform regression testing only with public releases of Geant4 (in other words, one Geant4 release per year). An extensive effort is under way to achieve this goal.

The regression testing showed that the hadronic physics component of Geant4 can change significantly from a Geant4 release to the next one and needs particular attention. Provided the complexity of this physics component, there is a need to extend the G4‐Med system to a wider set of tests dedicated to fundamental physics quantities (e.g., hadronic cross sections and final states of individual interactions). In addition, more tests should be included in the G4‐Med benchmarking system, especially for radioactivity, nuclear medicine, and neutron production and interactions.

## CONFLICT OF INTEREST STATEMENT

The authors declare no conflicts of interest.

## Footnotes

1 Results were obtained under ESA Contract 4000121062/17/NL/LF “Experimental Evaluation of ATHENA Charged Particle Background from Secondary Radiation and Scattering in Optics (EXACRAD).” Relevant contribution has been given by Bernard Dirassen, ONERA, and Thierry Paulmier, ONERA, for experimental measurements of electron emission yield and spectra; Paolo Dondero, SWHARD SRL, Fan Lei, RadMod Research Ltd., Alfonso Mantero, SWHARD SRL, for simulations and data analysis; Simone Lotti, INAF IAPS‐Roma, Silvano Molendi, INAF IASF‐Milano, for coordination and project management.
